# Investigation of SAMD1 ablation in mice

**DOI:** 10.1038/s41598-023-29779-3

**Published:** 2023-02-21

**Authors:** Bruce Campbell, Lisa M. Weber, Sandra J. Engle, Terence R. S. Ozolinš, Patricia Bourassa, Robert Aiello, Robert Liefke

**Affiliations:** 1 Atherex Inc., Lincoln, MA 01773 USA; 2 Institute of Molecular Biology and Tumor Research (IMT), Philipps University of Marburg, 35043 Marburg, Germany; 3 Department of Hematology, Oncology, and Immunology, University Hospital Giessen and Marburg, 35043 Marburg, Germany; 4 Biogen, Cambridge, MA 02142 USA; 5 Department of Biomedical and Molecular Sciences, Queen’s University, Kingston, ON K7L 3N6 Canada; 6 Cybrexa Therapeutics, Groton, CT 06340 USA; 7 Pfizer Inc., Groton, CT 06340 USA

**Keywords:** Developmental biology, Chromatin

## Abstract

SAM domain-containing protein 1 (SAMD1) has been implicated in atherosclerosis, as well as in chromatin and transcriptional regulation, suggesting a versatile and complex biological function. However, its role at an organismal level is currently unknown. Here, we generated SAMD1^−/−^ and SAMD1^+/−^ mice to explore the role of SAMD1 during mouse embryogenesis. Homozygous loss of SAMD1 was embryonic lethal, with no living animals seen after embryonic day 18.5. At embryonic day 14.5, organs were degrading and/or incompletely developed, and no functional blood vessels were observed, suggesting failed blood vessel maturation. Sparse red blood cells were scattered and pooled, primarily near the embryo surface. Some embryos had malformed heads and brains at embryonic day 15.5. In vitro, SAMD1 absence impaired neuronal differentiation processes. Heterozygous SAMD1 knockout mice underwent normal embryogenesis and were born alive. Postnatal genotyping showed a reduced ability of these mice to thrive, possibly due to altered steroidogenesis. In summary, the characterization of SAMD1 knockout mice suggests a critical role of SAMD1 during developmental processes in multiple organs and tissues.

## Introduction

Human SAMD1 (SAM domain-containing protein 1) is a 538 amino acid protein that has orthologs in most vertebrates, including zebrafish. Originally, SAMD1 was identified as a protein involved in atherosclerotic low-density lipoprotein (LDL) binding in humans^1^ and mice^2^. SAMD1 may participate in LDL retention and uptake, and its knockdown suppresses vascular smooth muscle cell (VSMC) differentiation and proliferation, which affects foam cell development^3^. However, SAMD1 is widely expressed in most organs and tissues, both in mice and humans (see ProteinAtlas, GTEx, etc.). The extensiveness of expression suggests roles in adult homeostasis, but the molecular function of SAMD1 has been minimally studied so far. SAMD1 was found to directly associate with CpG islands at the chromatin, acting as a transcriptional regulator, and be required for proper embryonic stem (ES) cell differentiation^4^. Earlier reports further support epigenetic functions for SAMD1. SAMD1 was enriched at nucleosomes and H3K4me3-possessing chromatin regions, as revealed by mass spectrometry^5,6^. It was also among the highest 5% of proteins that associate with both H3K4me3-modified and bivalently H3K4me3/H3K27me3-modified chromatin^7^.

The SAMD1 protein has two globular domains. At the C-terminus, it possesses a SAM domain that can interact with other SAM domain-containing proteins, such as L3MBTL3, and allows multimerization^4^. The N-terminal domain belongs to a group of related winged helix domains that bind to unmethylated CpG motifs^4,8^, explaining the enrichment of SAMD1 at CpG islands on the genome^4,9^. SAMD1 associates with repressive chromatin regulatory complexes that contain the histone demethylase KDM1A and several other SAM domain proteins, including the Polycomb group-related proteins L3MBTL3 and SFMBT1^4,10–12^. Consequently, the absence of SAMD1 typically leads to the derepression of its target genes^4,9^. SAMD1 is often upregulated in cancer tissues and correlates with worse prognosis in some cancer types, such as adenoid cystic carcinoma (ACC) and liver cancer^9,13^. A CRISPR screen in K562 cells suggested that SAMD1 is required for the efficient proliferation of these cells^14^. Further work demonstrated that SAMD1 deletion in HepG2 hepatocellular carcinoma cells leads to a global readjustment of the active H3K4me2 chromatin mark and a more favorable gene signature, supporting an oncogenic role in this context^9^. SAMD1 has also been described to play a role in muscle adaptation^15^. Taken together, these findings suggest that SAMD1 likely has a function in multiple biological processes and diseases, but the biological role of SAMD1 at the organismal level has yet to be investigated.

Here, we report the first knockout of SAMD1 in mice. Fewer homozygous (SAMD1^−/−^) embryos were observed at embryonic day (E) 14.5, and none were observed after E18.5. Development appeared grossly normal at E12.5, but at E14.5 the investigated embryos exhibited numerous defects, including degrading endothelial cell (EC) tubes, degrading inner organs and an almost complete absence of red blood cells (RBCs). The heterozygous (SAMD1^+/−^) mice had a less severe phenotype and approximately 70% of the mice survived past week 3 postnatally but they exhibited reduced weight gain and hormonal changes.

## Results

### SAMD1 knockout mice are embryonic lethal

**Figure 1:**
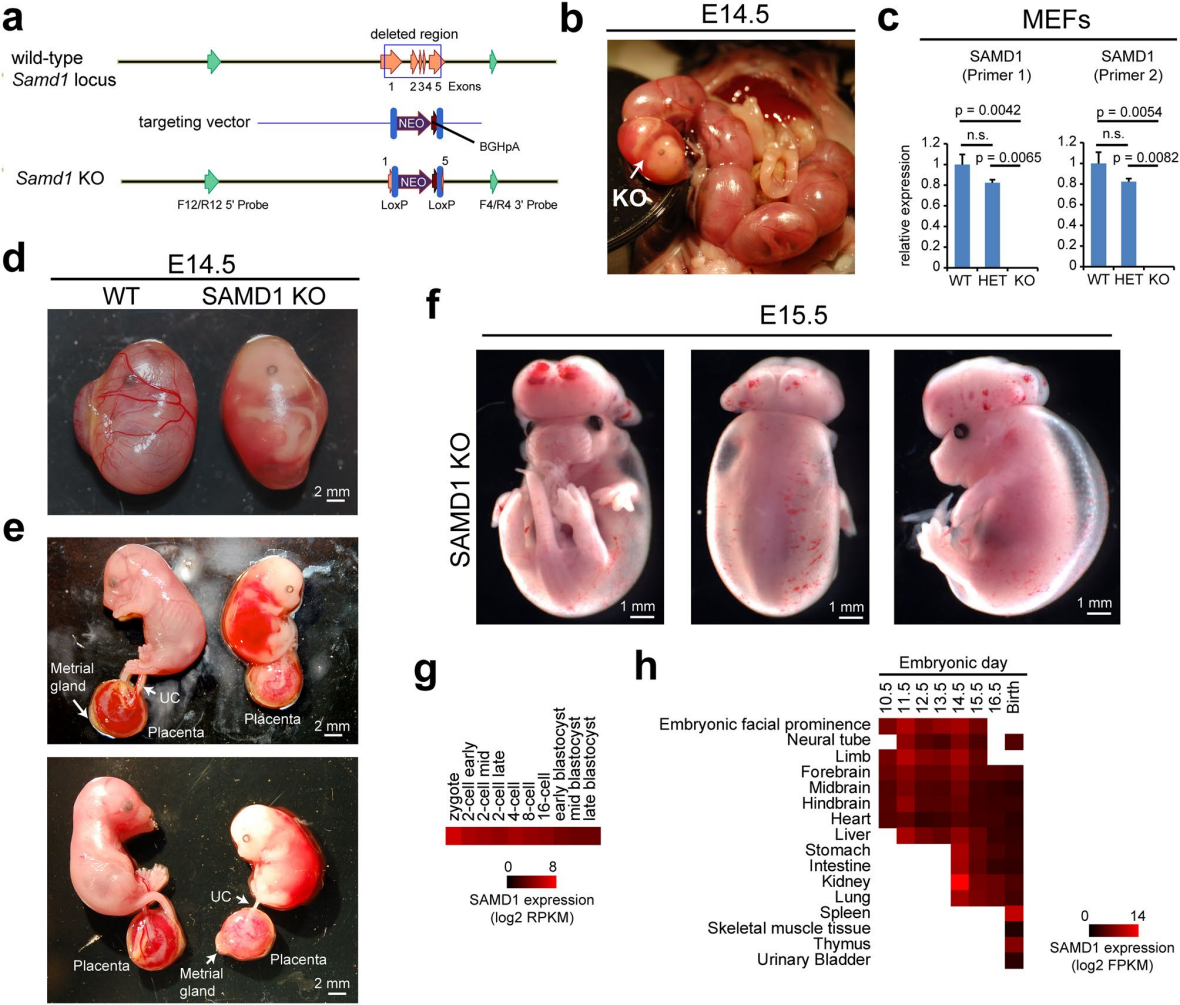
SAMD1 KO mice are embryonic lethal and lack intact blood vessels. (**a**) Gene targeting strategy to obtain SAMD1 KO mice. Southern probes are marked in green. (**b**) A SAMD1 KO embryo is easily distinguished from the WT embryos as soon as the litter is partially removed from the uterus. (**c**) RT-qPCR of SAMD1 in mouse embryonic fibroblasts (MEFs) obtained from wild-type and SAMD1 homo- and heterozygous embryos. Data are presented as the mean ± SD of two biological replicates. P-values via two-way ANOVA with Tukey’s multiple comparisons test. n.s. = not significant. (**d**) Pictures of embryos at E14.5 within the yolk sac. (**e**) Pictures of embryos at E14.5, without yolk sac, but still within the amnion. The placenta is shown underneath. (**f**) Picture of a SAMD1 KO mouse at approx. E15.5. (**g**) Gene expression of SAMD1 during early embryogenesis^23^. (**h**) SAMD1 gene expression in various embryonic tissues based on data from ENCODE^24^.

The SAMD1 gene was deleted from C57BL/6J mice by recombineering (Fig. 1a), which led to the removal of 2396 bp of the SAMD1 gene. Successfully obtained heterozygous mice were mated. The ratios between the wild-type (WT, aka SAMD1^+/+^), heterozygous (HET, aka SAMD1^+/−^), and knockout (KO, aka SAMD1^−/−^) mice were close to the expected Mendelian ratios through E12.5. All embryos removed at E12.5 had heartbeats. The first dead SAMD1 KO embryos and abnormal phenotypes were observed at E14.5 (Fig. 1b). At this time only 16 of the Mendelian expected 25 KO embryos were present, meaning that approximately 9 embryos had been resorbed since E12.5 (p = 0.13). Additional reductions in KO embryos manifested at later time points (Supplementary Table S1) (p = 0.049). Dying pups were not seen, so the few KO mice that had not been resorbed by E18.5 died just prior to birth (Supplementary Table S1). This suggests there was variable expression and penetrance, and thus multiple embryonic lethal SAMD1 KO phenotypes. Crossing SAMD1^+/−^ mice with SAMD1^+/−^ mice and phenotyping 3 weeks after birth (P21) also showed no KO (p = 5.1 × 10^–13^) (Supplementary Table S2), confirming the lethality of the SAMD1 KO.

In contrast, the E14.5 heterozygous (HET) embryos were not noticeably different from that of the WT embryos, and HET mice were born alive (Supplementary Table S1). This observation suggests that heterozygous deletion of SAMD1 had no or only a minor impact on embryogenesis. To understand the underlying reason, embryonic fibroblasts (MEFs) raised from WT, HET, and KO mice were investigated for SAMD1 expression via RT-qPCR. SAMD1 mRNA was slightly but not significantly reduced in the heterozygous cells and absent in the homozygous KO cells (Fig. 1c). The mild reduction in SAMD1 expression in the heterozygous cells raises the possibility that a feedback mechanism at the wildtype allele compensates for the loss of SAMD1 expression from the knockout allele^16^. This mechanism could be the underlying reason for the mostly normal development of the heterozygous mice. However, genotyping of the surviving mice at P21 showed that the number of observed HETs was significantly lower than the expected number, implying that the heterozygous mice also have some impairments. Reduced numbers of HET mice at P21 were observed both when SAMD1^+/−^ mice were crossed with SAMD1^+/−^ mice (p = 0.003) (Supplementary Table S2), and when SAMD1^+/+^ mice were crossed with SAMD1 ± mice (p = 0.003) (Supplementary Table S3).

### Defects of the SAMD1 KO embryos are visually obvious

Given the considerably severer impairment of KO embryogenesis compared to HET mice, we first analyzed the KO embryos in more detail. We focused on E14.5 embryos for further investigations, because the E12.5 KO mice appeared normal, and E14.5 has been established as an optimal time point for mouse developmental disorder phenotype analyses^17,18^. Macrolevel photography of the E14.5 WT embryos within the yolk sac showed normal development, noting branching blood vessels in the yolk sac (Fig. 1b,d). In contrast, the yolk sac of the KO embryos lacked obvious blood vessels, it had only a few broken thin red lines, and the amniotic fluid was pink as if blood had leaked into it (Fig. 1b,d). In the KO mice the yolk sac did not seem to be normally attached to the placental disk, suggesting initiation of the abortion process.

When the yolk sac was removed, the KO embryo within the amnion appeared developmentally delayed and visually smaller than the WT embryo (Fig. 1e). Bloody fluid was also pooled around the midsection within the amnion, but the source of leakage was not obvious (Fig. 1e). The embryo surface was very pale, and across the back, clear edema could be seen separating the skin from the tissue beneath. Red blood cells were not obvious in the skin, other than on the head and back, where a few small hemorrhages and a few broken red lines that were probably failed blood vessels were recognizable. This observation may suggest abnormal vessel regression. Regression, also called “pruning”, is a normal part of embryonic maturation of blood vessels^19,20^. It can include pruning of individual EC (endothelial cell) tubes, as well as complete regional network regression, leaving behind "sleeves” of collagen and apoptotic ECs^21^. During maturation, progenitor/pericyte/smooth muscle cells connect to EC tubes, providing stability needed to control regression^20^. The presence of RBCs in the KO skin implies that a functional circulatory system existed prior to an apparent abnormal regression of the EC tube vascular plexus in the KO embryo.

Similar to the embryo, the KO placenta was pale compared to the WT (Fig. 1e), probably because the labyrinth contained fewer RBCs and the vasculature seemed to be pink instead of red. The metrial gland lacked obvious blood vessels and the decidua appeared to be thinner. Maternal blood vessels could be seen in a few locations immediately beneath the decidua. The umbilical cord was avascular, showing cessation of maternal-embryo blood flow (Fig. 1e).

At E15.5 (Fig. 1f), a SAMD1 KO embryo lacked a skull vault, had obvious exencephaly, and had a hypertrophic brain. Clear edema fluid separated the skin from most of the embryo surface. This kind of edema appears in lymph endothelial cell progenitor knockouts^22^. The E15.5 embryo was less pale than the E14.5 embryo, had longer and larger red lines, and had a few apparently intact blood vessels. Some areas of the embryo surface in and beneath the skin, and above the brain, had small hemorrhages and lines of RBCs that may have once been contained in vessels, suggesting a failing circulatory system. Since the E14.5 KO had a skull vault and was paler, the investigated E15.5 KO embryo is likely an example of a different SAMD1 KO phenotype, but the surface RBC patterns appear similar to the E14.5 KO. Failed skull vault development may be caused by failure of neural tube closure. Clear fluid leakage due to failing lymphatic and blood vessels likely produced edema (Fig. 1f). Both vessel types are constructed from CD31+/VEGFR2+ ECs, suggesting a common point of failure.

Given the strong defects observed in SAMD1 KO embryos, we used public gene transcription data to assess embryonic SAMD1 expression^23,24^. SAMD1 mRNA was detected in very early developmental stages (Fig. 1g) as well as in all investigated organs during all embryonic stages (Fig. 1h). Similar results were also obtained using RT-qPCR experiments from isolated embryonic organs (Supplementary Fig. S1). This observation suggests that SAMD1 has a biological function during all embryonic stages and in most embryonic tissues.

### SAMD1 KO embryos have multiple organ defects

**Figure 2:**
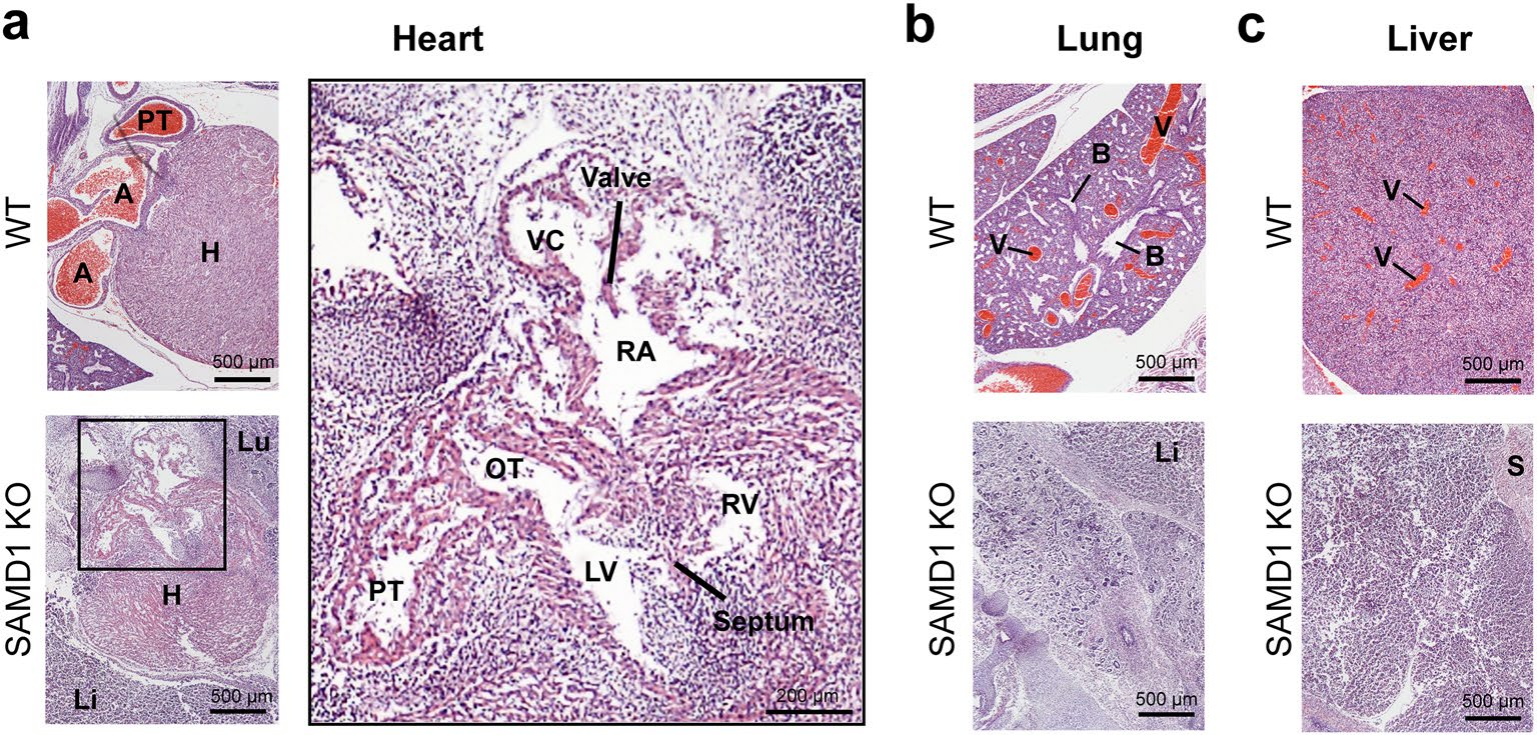
SAMD1 KO embryos exhibit organ degradation. (**a**) H&E staining of the heart at E14.5. *H* heart muscle, *A* aorta, *PT* pulmonary trunk, *Lu* lung, *Li* liver, *RA* right atrium, *OT* outflow tract of left ventricle, *VC* vena cava, *LV* left ventricle, *RV* right ventricle. (**b**) H&E staining of the lungs at E14.5. *B* bronchiole, *V* blood vessel, *S* skeletal muscle. (**c**) H&E staining of the liver at E14.5. *V* blood vessel, *S* skeletal muscle.

To further assess the consequence of the SAMD1 KO on specific organs, we first used hematoxylin and eosin (H&E) staining on sagittal slices of an E14.5 embryo (Fig. 2). Hematoxylin reveals not only nuclei, but also glycosaminoglycans, for example in cell walls, and thus allows visualization of fragmenting cells, while eosin stains cytoplasm and most connective tissue pink, orange, and/or red^25^. A comparison of embryonic tissues and organs in WT, HET and KO mice revealed several abnormalities in the KO mice, while the HET embryo organs were indistinguishable from the WT organs (Fig. 2, Supplementary Fig. S2). Although cardiac muscle cells in the KO embryo stained red and had associated nuclei, the heart appeared to be breaking up, and the atria, ventricles, and pulmonary trunk had partially collapsed (Fig. 2a, right panel). The endocardium was separating from the chamber walls. Red-stained RBCs were absent, and the ventricles appeared to contain some lymphocytes/immune cells instead of RBCs (Fig. 2a, right panel). This suggests RBCs were trapped due to ejection failure and became necrotic.

The KO lung also appeared to be fragmenting, and RBCs were not apparent, in contrast to the WT lung where RBCs filled large vessels (Fig. 2b). Bronchioles had formed but were smaller than those in the WT. They appeared to be degrading and may not have formed correctly. Faint pink staining of connective tissue in the lung and diaphragm may indicate dying cells.

The KO liver had lobes and was large enough to have developed at least until E12.5 (Fig. 2c). Similar to the heart, the liver had no RBCs, again in contrast to the WT, where RBCs filled large vessels. The tissue was broken up in patterns that suggested degradation rather than a malformation. A possible portal triad is visible, but other features were not readily identifiable. The fact that organs were substantially developed means that a functional circulatory system necessarily existed prior to E14.5.

### SAMD1 deletion led to disorganization and reduced levels of RBCs in the skin

The most obvious difference between the appearances of WT and KO embryos was the paleness of the latter, suggesting impaired blood circulation. Thus, we tested an E14.5 embryo for the presence of endothelial cells (ECs), which line all blood and lymph vessels, from primitive endothelial tubes to fully mature vessels. We used H&E, CD31 or VEGFR2 staining (with hematoxylin counterstain) to mark ECs and provide morphological identification of other cells. Cluster of differentiation 31 (CD31, also known as PECAM1) is widely used as a marker for ECs, staining the cell surface. Vascular endothelial growth factor 2 (VEGFR2, also known as FLK1) stains the cytoplasm of ECs. Using these different markers, we investigated the organs and tissues of the SAMD1 KO embryos.

**Figure 3:**
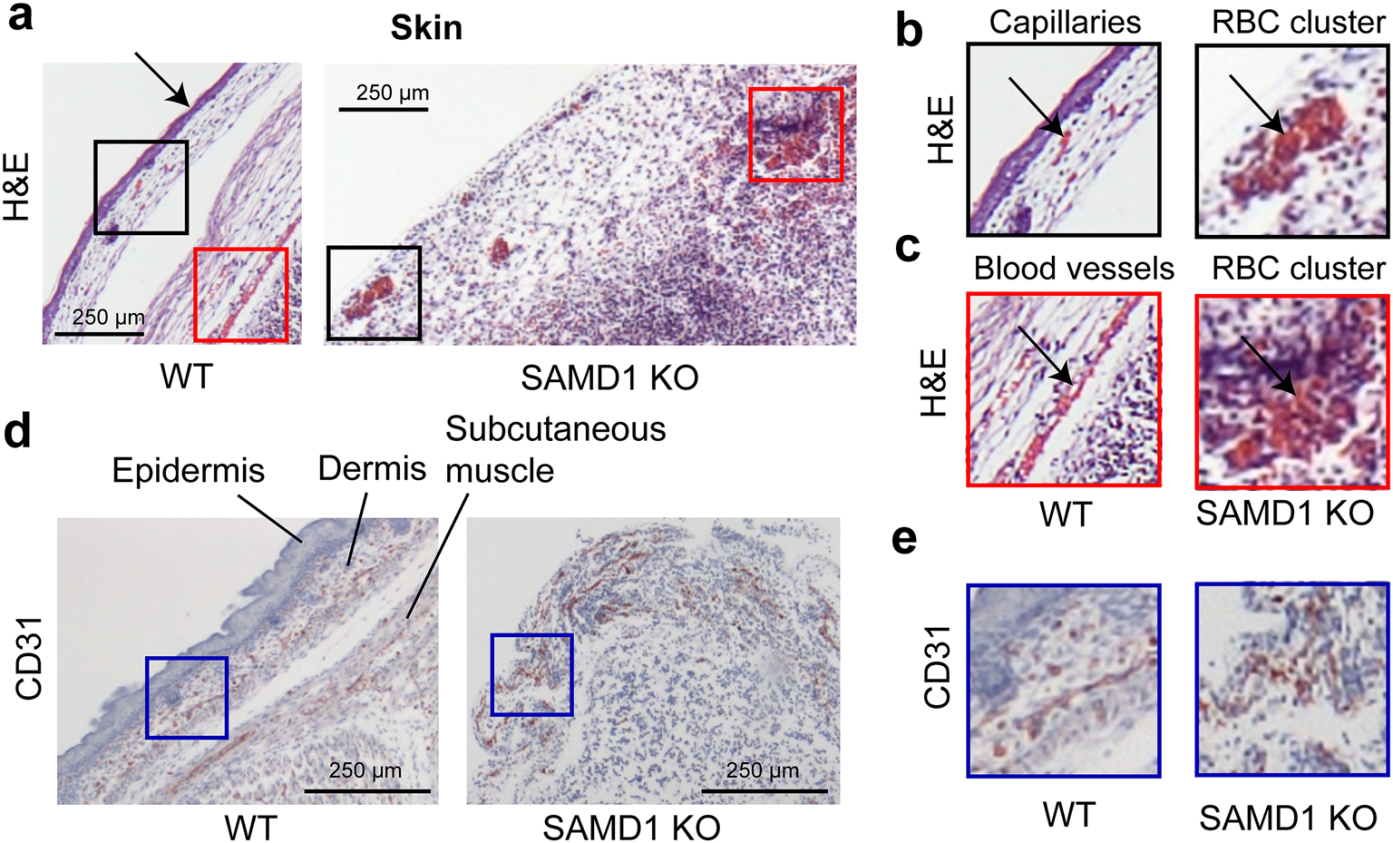
E14.5 SAMD1 KO mice have clusters of RBCs and disorganized and damaged endothelial cells (ECs) in the skin. (**a**) H&E staining of embryo skin. (**b**) Higher magnification showing capillaries. Arrows indicate capillary (WT) or RBC clusters (KO). (**c**) Higher magnification of blood vessels (WT) or RBC clusters (KO) are indicated by arrows. (**d**) CD31 (brown) staining of embryo skin (**e**) Higher magnification of CD31 ECs in capillaries in WT skin and disordered structures in KO skin.

Looking first at the embryo surface, H&E staining of the WT embryo showed groups of RBCs in the skin and RBCs in a larger blood vessel beneath the subcutaneous tissue. Here, CD31 stained ECs of capillaries and larger vessels (Fig. 3a–c), as expected. In contrast, KO skin, except around the limbs and skull, appeared to have been lost during fixation (Fig. 3a), likely due to edema separating the skin from the tissue beneath. In locations beneath the lost skin, the KO embryonic surface appeared to consist of degrading and fragmenting skeletal muscle (Fig. 3a,d). This tissue included individual RBCs and a few scattered clusters of RBCs that, lacking typical organizational patterns, did not appear to be contained in vessels (Fig. 3a–c). Coagulation in the clusters could not be determined, but gradations in red staining implied the recent onset of RBC degradation. These extravascular RBCs could be the result of fresh microhemorrhages or RBCs recently stranded as vessel development failure halted circulation. Consistent with this second hypothesis, intact vessels were not obvious, but RBCs could not have arrived at their present locations unless at least functional endothelial tubes had once been present. CD31 stained numerous misshapen subcutaneous ECs in broken brown lines (Fig. 3e). These patterns suggested that the ECs were part of capillaries or larger vessels that had degraded. Cytoplasmic instead of cell surface CD31 brown staining patterns suggest necrosis, and the association of CD31 staining with rounded nuclei suggests possible phagocytosis of ECs. Several broken small circles of stained ECs also suggested failed vessels (Fig. 3d,e).

Edema did not separate the skin from the KO forepaws, which appeared to have developed normally except for being smaller, pale, and lacking intact blood vessels (Supplementary Fig. S3). H&E staining of the forepaw showed a small group of faintly stained RBCs, where faint blurred hematoxylin stain suggested impending necrosis. CD31 staining showed a few nuclei partially surrounded by brown-stained EC material, again suggesting ingestion. The patterns of broken lines suggest the presence of failed small vessels in the forepaw (Supplementary Fig. S3, arrows). Interestingly, red-stained RBCs were noted primarily in skeletal muscle near the embryo surface but not in the heart or other organs. Surface perfusion may have been sufficient to delay RBC necrosis compared to locations deeper in the embryo.

### SAMD1 KO embryos have degraded internal organs and blood vessels

**Figure 4:**
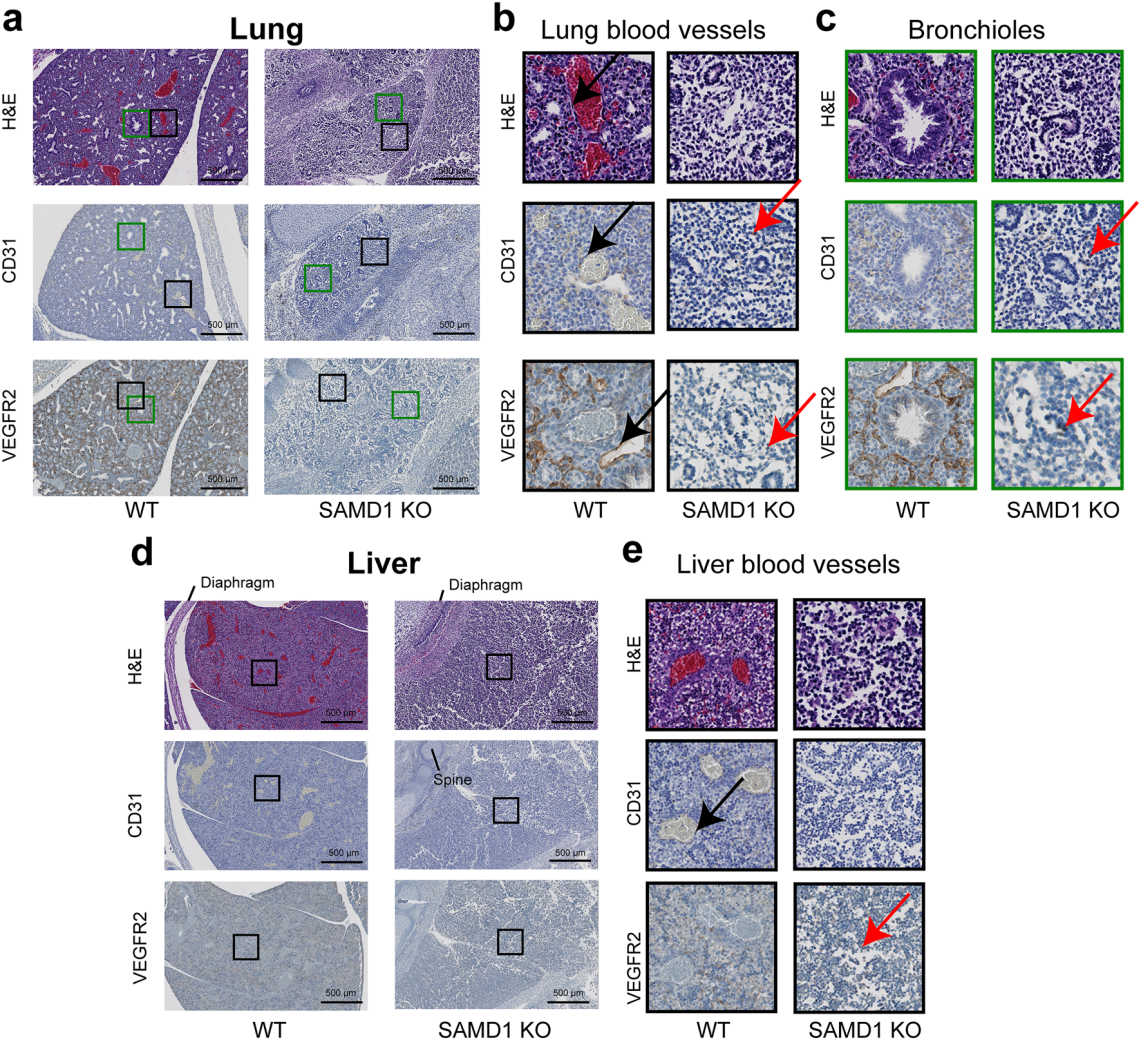
Absence of blood vessels in the lung and liver of SAMD1 KO embryos. (**a**) Macroscale microscopy of the lungs from wild-type and SAMD1 KO mice at E14.5 using H&E, CD31 (brown), and VEGFR2 (brown) staining. (**b**) Higher magnification of lung wild-type blood vessels; the black arrows point to spindle-shaped vascular smooth muscle cells (VSMCs) adjacent to ECs, that are not present in the KO lung. (**c**) Higher magnification of bronchioles, which are smaller and/or degraded in the KO mice. The lung appears to be fragmenting along the paths of failed vessels. (**d**) Macroscale microscopy of the lungs from wild-type and SAMD1 KO mice at E14.5 using H&E, CD31, and VEGFR2 staining. (**e**) Higher magnification of blood vessels in the wild-type liver, and their absence in the KO. The KO liver also appears to be fragmenting. Red arrows in b, c, and e indicate possible fragments of phagocytized cells.

**Figure 5:**
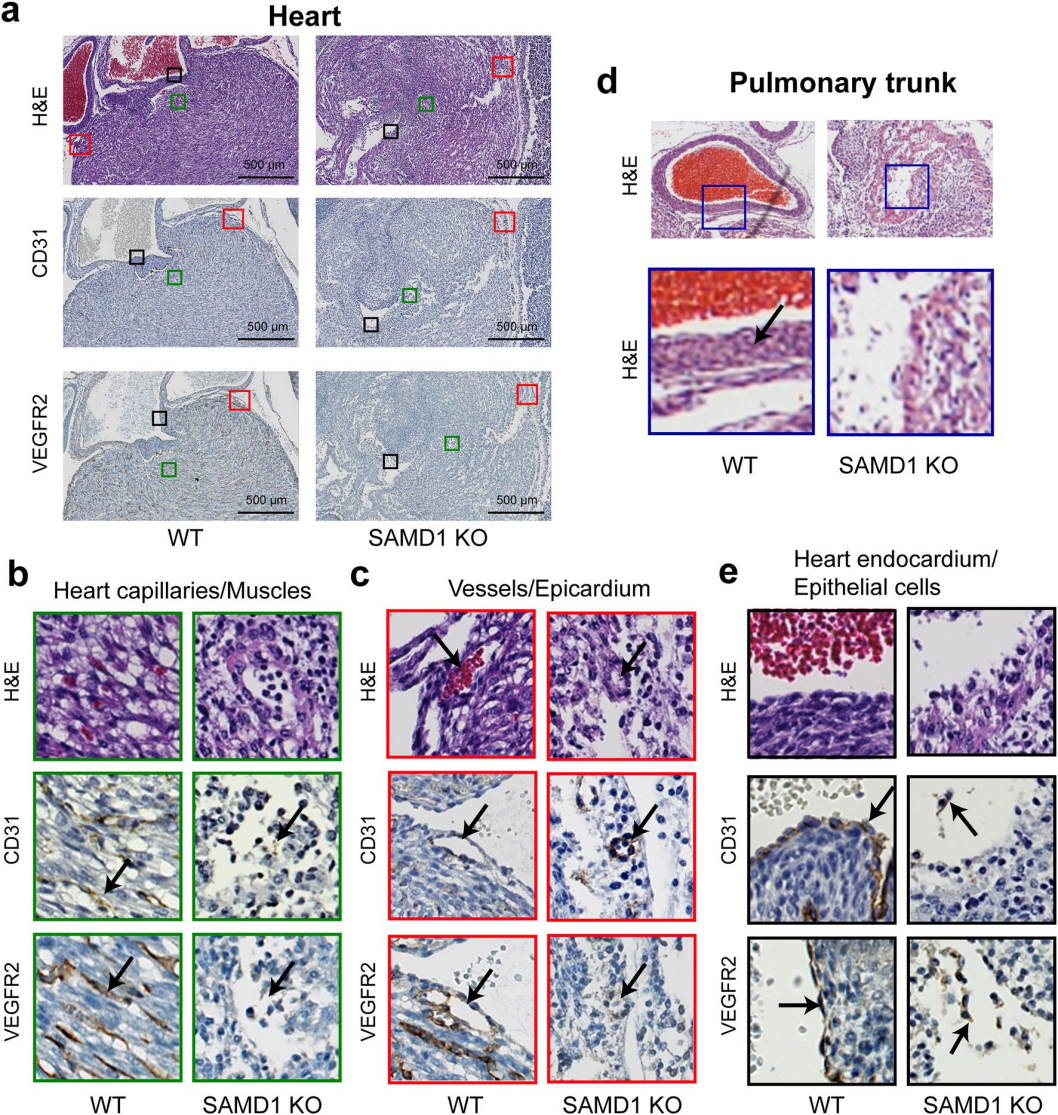
SAMD1 KO mouse hearts are fragmenting, capillaries are missing, and larger vessels and the epithelial layer are failing. (**a**) Microscopy of the heart using H&E staining as well as staining for CD31 and VEGFR2 at E14.5. (**b**) Higher magnification comparing WT with myocardium capillaries to KO missing myocardium capillaries. The arrows indicate intact and degraded ECs; note degraded myocardium. (**c**) Higher magnification of an intact WT compared to a degrading KO coronary vessel beneath the epicardium. The arrows indicate intact and degraded coronary vessel ECs. (**d**) H&E staining of the pulmonary trunk from WT and SAMD1 KO mice at E14.5 (derived from same embryo as in Fig. 2a). Instead of spindle-shaped cells (blue), marked with an arrow in the WT, a compact myocardium lines the SAMD1 KO pulmonary trunk. (**e**) Higher magnification of heart endocardium and epithelial cells. The arrows indicate intact (WT) and degraded (KO) epithelial cells (brown).

Our initial analysis (Fig. 2) suggests that internal organs are fragmented and that they lack proper blood vessels. To further assess the impact of SAMD1 deletion on blood vessels in these internal organs, we stained lung, liver (Fig. 4), and heart samples (Fig. 5) from E14.5 WT and KO mice with H&E, CD31, or VEGFR2. In all WT samples, H&E staining showed normal organ development and large blood vessels filled with RBCs (Figs. 4, 5). Larger lung and liver vessels, identified by sizeable areas of RBCs, were encircled by stained ECs (Fig. 4b,d). Capillaries were also made evident by the presence of EC markers and RBCs. Pulmonary vascular ECs normally line the surfaces of the lung vasculature, which stained strongly for CD31 and VEGFR2, and epithelial cells in bronchioles stained faintly for VEGFR2 (Fig. 4c). Liver sinusoidal ECs also stained for CD31 and VEGFR2 (Fig. 4e).

In contrast, in the lungs and livers of the SAMD1 KO mice, CD31 and VEGFR2 staining was almost absent. In KO organs, no RBCs or intact ECs were visible (Fig. 4a,b,d,e). CD31 and VEGFR2 stained vessels that should have surrounded alveoli were absent (Fig. 4c). The limited CD31 and VEGFR2 staining in the lung and liver appeared to be cell fragments of necrotic vessel wall ECs, and the thin lines of associated faint hematoxylin stain likely mark dermatan sulfate and heparan sulfate from these ECs. Examples of probable phagocytosis in the liver were indicated by uneven brown staining around strongly blue-stained round nuclei (Fig. 4b,c,e, red arrow, Supplementary Fig. S4a). This implies that ECs had been present, became necrotic and were being removed. Bronchioles had formed in the KO lungs but were much smaller than those in the WT lungs (Fig. 4c). Based on the available data, it is not possible to judge whether the smaller bronchioles in the KO were due to incomplete formation during development or whether they had degraded (Fig. 4c). Malformed and/or underdeveloped liver lobules and tubes appear to be degrading (Fig. 4d).

In the WT hearts, normal CD31 and VEGFR2 staining patterns were noted in ECs of capillaries and larger vessels (Fig. 5a–c). H&E staining of the WT pulmonary trunk showed numerous RBCs contained in the vessel, demonstrating normal heart function. VEGFR staining also identified ECs at the lumen and several layers of circumferentially oriented spindle-shaped vascular smooth muscle cells (VSMCs) (Fig. 5d). As expected, epithelial cells in the endocardium stained for CD31 and VEGFR2 (Fig. 5e).

In the KO heart, H&E staining uncovered signs of epithelial, cardiac muscle cell, and EC degradation (Figs. 2a, 5b). Hematoxylin counterstain in the CD31 and VEGFR2 slides revealed fragmenting cardiac muscle cells (Fig. 5b). In a few places, round hematoxylin-blue nuclei were loosely associated with faint CD31 or VEGFR2-brown patterns demonstrating the presence of necrotic ECs and epithelial cells (Fig. 5c). Phagocytosis in the heart was indicated by uneven and foamy brown staining around strongly blue-stained round nuclei (Supplementary Fig. S4b). CD31 and VEGFR2 stained broken circles and lines of misshapen ECs and EC fragments. These patterns suggested incomplete and/or failed vessels (Fig. 5b,c). This was seen deep in the heart muscle where capillaries should have been (Fig. 5b), and more frequently at the heart’s surface, where larger stained circles suggested failed coronary vessels (Fig. 5c). Spindle-shaped VSMCs (blue) that were noted next to coronary artery ECs in the WT were absent from the KO (Fig. 5e). The KO pulmonary trunk wall consisted only of the myocardium, with a few disconnected ECs above the lumen, and lacked the VSMCs that were seen in the WT (Fig. 5a,d). The lumen of the KO pulmonary trunk contained no RBCs and only a few rounded probable immune cells (Fig. 5d). The heart chambers and large vessels were partially collapsed, possibly due to serum fluid being insufficient to maintain shape (Figs. 2a, 5a–c). The ventricles contained dark blue stained rounded probable immune cells and cell fragments that may have been RBCs, but trabeculation was not observed. Epithelial and endothelial cells are closely linked. During embryonic development, the epithelial layer is the source of endothelial cells for the heart’s blood vessels^26^. CD31- and VEGFR2-stained epithelial cell fragments were detectable in the KO endocardium, including detached cells and cells undergoing probable necrosis and phagocytosis (Fig. 5e). These above findings suggest that in the investigated SAMD1 KO mouse the development of the heart may have stopped before VSMC differentiation.

Together our analysis of internal organs using markers for ECs suggests that the blood vessel system was degrading and non-functional at E14.5, leading to organ degradation.

### SAMD1 KO ribs showed premature ossification

**Figure 6:**
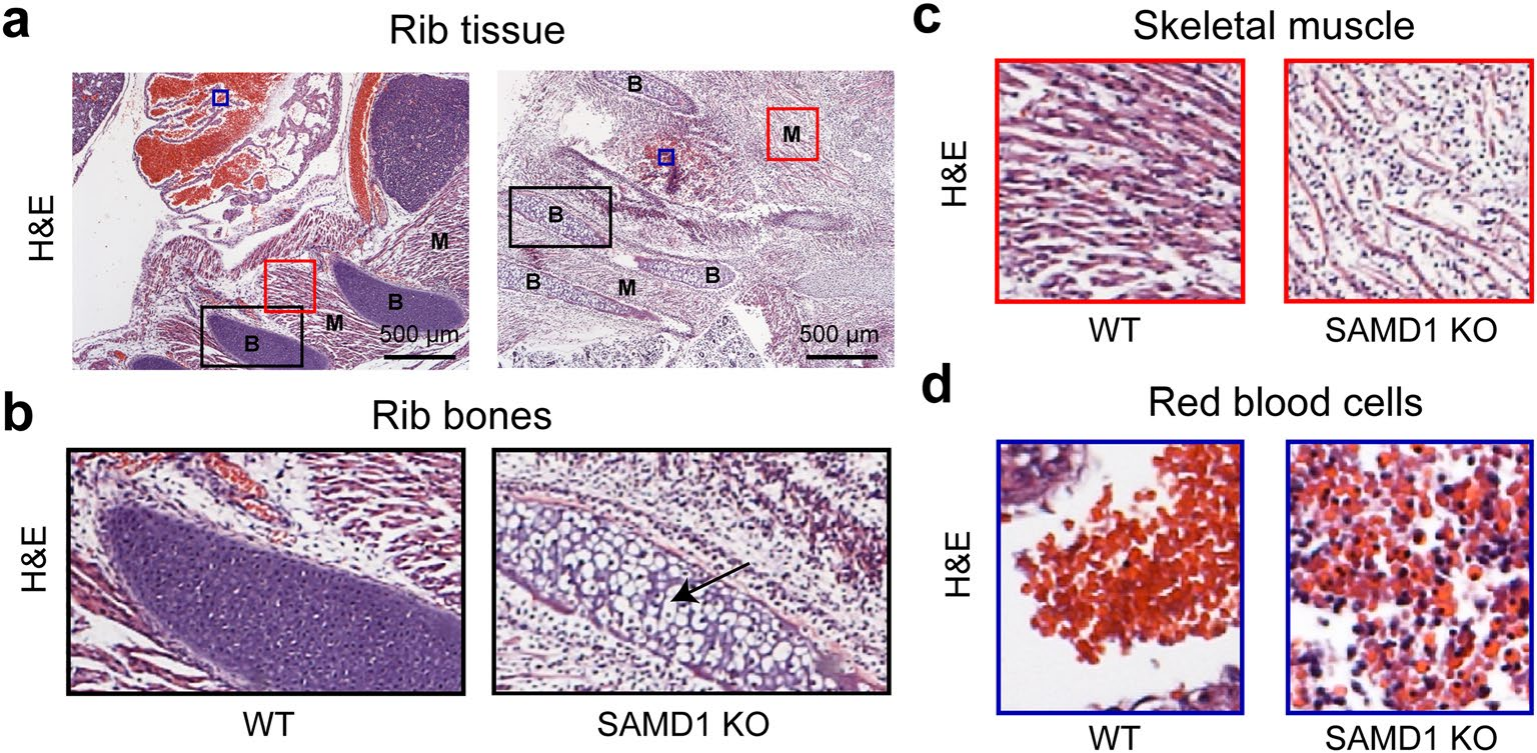
The absence of SAMD1 led to early bone ossification, muscle degradation, and the enduring presence of nucleated RBCs. (**a**) Macroscale microscopy of embryonic rib tissue using H&E staining at E14.5. *B* bone, *M* muscle. (**b**) Higher magnification of the rib bones, showing cartilage primordium (purple) in the wild-type mice and ossification (bubbly, indicated by the arrow) in the KO mice. (**c**) Higher magnification of the skeletal muscles shows separated and fainter muscle fibers (pink) in the KO and more unstained tissue. (**d**) Higher magnification of H&E staining of red blood cell pools from WT and SAMD1 KO embryos at E14.5 shows nucleated RBCs and immune cells in the KO.

Next, we investigated the ribs and associated skeletal muscles. Structural organization in the E14.5 WT and KO mice were roughly similar (Fig. 6a), but H&E staining highlighted substantial differences. The WT rib consisted of cartilage primordium, while in the KO mouse, the rib was ossifying, as seen by the bubbly appearance of hypertrophic chondrocytes, some of which lacked nuclei (Fig. 6b, arrow). Bone development is tightly linked to hypoxia^27^, which is required for the transition from proliferating cartilage to endochondral ossification. Hypoxia interior to the developing bone causes chondrocytes to become hypertrophic and delays chondrocyte apoptosis/necrosis. Subsequent apoptosis/necrosis is required for ossification^27^. Chondrocytes in a vertebrae stain for VEGFR2 (Supplementary Fig. S4c), a receptor for VEGF, which is a chondrocyte survival factor necessary for bone formation^28^. Thus, we hypothesize that in the SAMD1 KO mice, the hypertrophic chondrocytes appeared to have become hypoxic before E14.5, instead of the normal condition at approximately E18, possibly due to the lack of RBC delivery.

Skeletal muscle cells in the WT mouse stained strongly with H&E and had associated blue nuclei (Fig. 6c). Strands of cells are separated by lines of unstained connective tissue (Fig. 6c). In the KO, there are several signs of degradation. The KO has fewer eosin-stained red strands, which are less strongly stained red and are more widely separated from each other than in the WT (Fig. 6c). The strands are also interspersed with many nuclei. Some of these nuclei may be immune cells that are attracted to necrotizing muscle cells. Hypoxia from a lack of delivery of RBCs is the likely cause of muscle cell necrosis.

Most RBCs (bright orange) in a blood vessel above a WT rib do not have nuclei, and few if any lymphocytes are seen (Fig. 6d). RBCs are absent from the KO, other than a few scattered pools near the embryo surface and near ribs (Fig. 6a). A small blood pool in the skeletal muscle above a KO rib contains some RBCs with eccentric nuclei and many lymphocytes/immune cells (rounded, blue) (Fig. 6d). Skeletal muscle cells immediately adjacent to this pool appears to be less degraded. The initiation of heartbeat around E8.5 marks the onset of embryo-vitelline circulation^29^, as yolk sac-derived hematopoietic cells are spread through the developing embryo^30^. These primitive erythroid (EryP) cells have nuclei, and begin to mix with definitive RBCs from approximately E12.5, and enucleating EryPs at E12.5^31^. A mix of nucleated and definitive RBCs remains in circulation until gestation. Obviously, nucleated and enucleated RBCs could not have arrived at these locations unless a functional circulatory system existed later than 12.5. This observation suggests circulation failure trapped EryPs and definitive RBCs in a nonfunctioning vessel around E13.5, when approximately 50% of RBCs would not have nuclei^31^. Some KO RBCs appeared faded (Fig. 6d), implying a recent loss of O_2_ from hemoglobin, and faint pink staining between RBCs and lymphocytes/immune cells suggests phagocytosis of RBCs.

Taken together, our work so far suggests that the absence of SAMD1 leads to improper development of all investigated organs and tissues in the embryo. We have summarized our findings in Table 1.

**Table 1:** Summary of defects observed in SAMD1 KO embryos.

Organ/tissue	Observed phenotype
Whole embryo	
E14.5	Pale Smaller Intact blood vessels not obvious
E15.5	Pale Some embryos show exencephaly, with lack of skull vault Intact blood vessels not obvious Edema fluid beneath skin scattered small blood pools and RBC clusters
Extra-embryonic (E14.5)	
Placenta	Pale
Yolk sac	Lacks blood vessels
Amnion	Pink amnionic fluids
Organs (E14.5)	
Blood cells	Extra-vascular, primarily in subcutaneous muscle, and adjacent to bone Pooled and scattered Excess of nucleated RBCs
Heart	Heart chambers collapsing; devoid of RBCs Lymphocytes/immune cells in chambers Muscle cells, epithelial layer, capillary ECs, and larger vessel ECs are degrading Broken lines of CD31+ and VEGFR2+ cells and degrading cells, indicate failed vessels
Liver	Malformed and/or underdeveloped lobules and tubes are degrading RBCs and blood vessels are absent Scattered clusters of CD31+ cells, CD31+ degrading cells, and very faint VEGFR2, indicating degrading blood vessels
Lung	Malformed and/or underdeveloped bronchioles are degrading RBCs and blood vessels are absent Scattered clusters of CD31+ cells and very faint VEGFR2, indicating degrading blood vessels
Skeletal muscle	Sparse muscle fibers, clusters of RBCs Absence of intact capillaries and larger vessels, indicated by broken lines of CD31+ and VEGFR2+ cells and degrading cells
Bone	Premature ossification; VEGFR2+ chondrocytes/osteoblasts Scattered CD31 and VEGFR2 stained material adjacent to bone, indicating degrading blood vessels
Paw	Several scattered RBCs; broken lines of CD31+ cells, indicating failing capillaries

### SAMD1 is required for proper neuronal differentiation in vitro

To better understand the molecular source of the many defects upon SAMD1 deletion, we investigated our previously published RNA-Seq data upon undirected ES cell differentiation in the absence of SAMD1^4^. Although SAMD1 functions mainly as a transcriptional repressor, we found both up and downregulated pathways upon SAMD1 deletion, suggesting direct and indirect consequences of SAMD1 deletion. Thus, it appears that SAMD1 plays a rather pleiotropic role during differentiation processes, consistent with the multifaceted mouse phenotype.

Our previous work showed that direct SAMD1 target genes are commonly associated with brain related pathways^4^ and several genes related to brain development, such as *Cbln1*, *Ntrk2*, and *Plxna4*, *Eph4*, become derepressed upon SAMD1 deletion during differentiation (Supplementary Fig. S5a). Consequently, pathways that are linked to brain development, such as synapse assembly (Supplementary Fig. S5b), become predominantly upregulated in SAMD1 KO cells upon differentiation^4^, suggesting that SAMD1 absence may be involved in regulating neuronal differentiation processes.

In contrast, pathways related to angiogenesis were mostly downregulated (Supplementary Fig. S5c). Intriguingly, vascular endothelial growth factor A (*Vefga*), *Pecam1* (CD31), *Thy1* and *Tie1*, which are critical factors for angiogenesis^32–34^, were strongly downregulated in SAMD1 KO cells, both in differentiated and undifferentiated cells (Supplementary Fig. S5d). Furthermore, pathways related to cardiac chamber development were also significantly dysregulated in differentiated SAMD1 KO cells (Supplementary Fig. S5e). This includes key transcription factors such as *Gata4*, *Gata6*, *Zfmp2* (*Fog2*) and *Mef2c*^35^ (Supplementary Fig. S5f). Thus, dysregulation of these genes and pathways during embryogenesis in the absence of SAMD1 could potentially contribute to the observed failure of angiogenesis and heart development. However, it is probable that angiogenesis would also fail upon the failure of arteriogenesis. Although significant research is ongoing, factors involved in arteriogenesis are still poorly understood compared to angiogenesis^36^.

**Figure 7:**
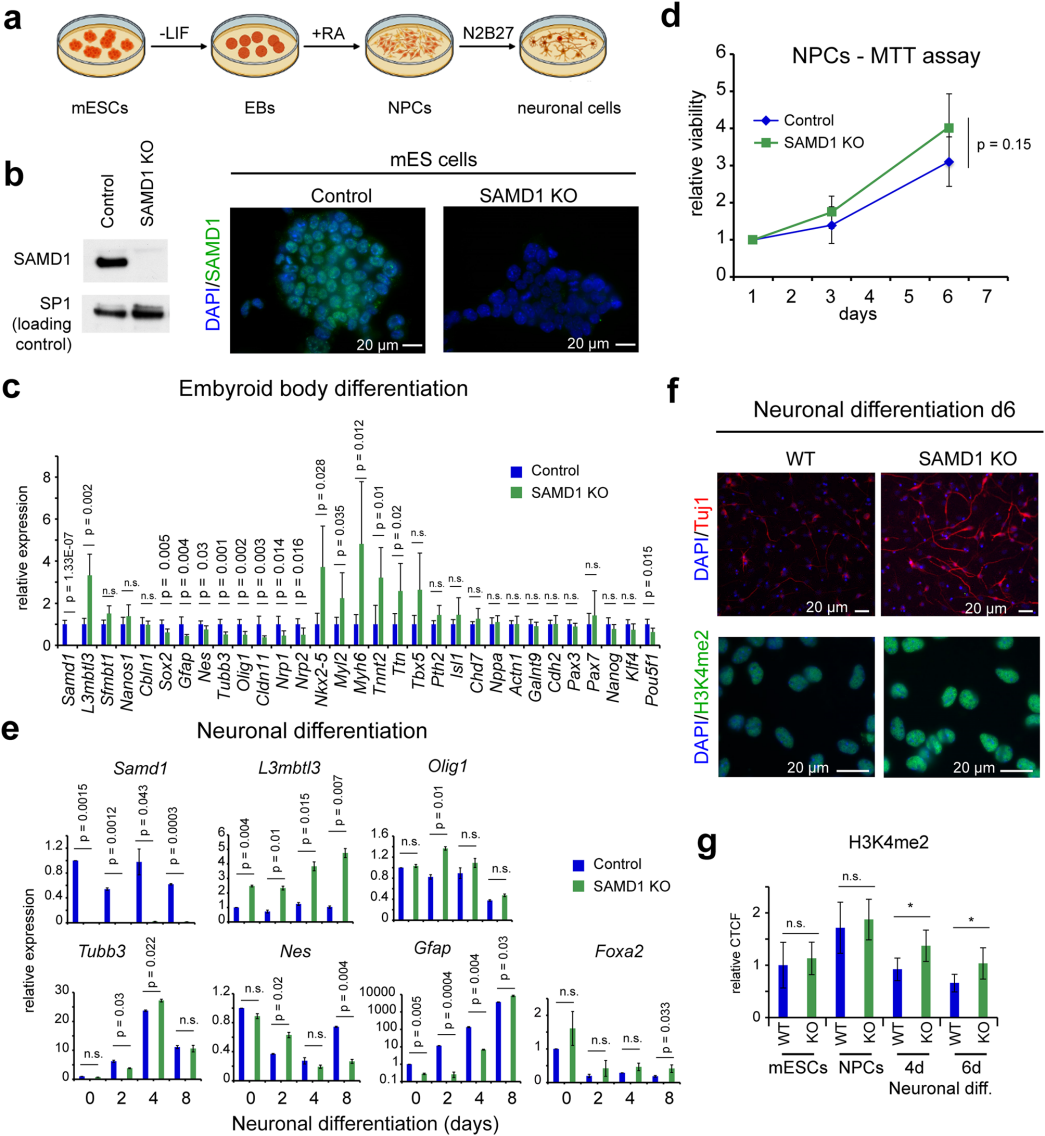
SAMD1 KO affects neuronal differentiation in vitro. (**a**) Schematic steps of the neuronal differentiation procedure. The graphic was created on BioRender.com. (**b**) Representative immunofluorescence and Western blots of wild-type (Control) and SAMD1 KO ES cells, which were established previously^4^. Full Westerns blots are shown in Supplementary Fig. S6. (**c**) RT-qPCR of differentiation-related genes after embryoid body formation. Data are presented as the mean ± SD of two biological replicates. (**d**) MTT assay of neuronal progenitor cells. Data are presented as the mean ± SD of three biological replicates. (**e**) RT-qPCR of marker genes upon neuronal differentiation, starting from NPCs. Data are presented as mean ± SD of two biological replicates. (**f**) Representative immunofluorescence for Tuj1 (Tubb3) and H3K4me2 in differentiated neuronal cells. (**g**) Quantification of H3K4me2 levels at distinct stages during the neuronal differentiation procedure. CTCF = corrected total cell fluorescence. Data are presented as mean ± SD of at least 15 cells per condition. Significance in (**c**, **d**, **e**, **g**) was evaluated via a two-tailed unpaired Student’s t-test. *n.s.* not significant.

Next, we wanted to gain more insights into whether ablation of SAMD1 can indeed influence specific differentiation processes. Direct SAMD1 targets are enriched at brain-related genes^4^, and the absence of SAMD1 leads to the upregulation of brain related pathways (Supplementary Fig. S5a,b). In contrast genes of other dysregulated pathways, such as angiogenesis, are not commonly targeted by SAMD1^4^, suggesting a more indirect influence of SAMD1 on these genes. Thus, we reasoned that neuronal pathways are more likely directly regulated by SAMD1, making them attractive for further investigation. Mouse ES cells can be differentiated into various lineages using specific protocols^37^, including neurons (Fig. 7a), offering us a starting point to investigate the role of SAMD1 in this process. Using our previously established SAMD1 KO mouse ES cells (Fig. 7b)^4^, we first created embryoid bodies, which already showed dysregulation of multiple genes (Fig. 7c). The embryoid bodies were differentiated into neuronal progenitor cells (NPCs) and subsequently into neuronal cells (Fig. 7a). We observed no significant differences in the cell growth of the NPCs, although SAMD1 KO NPCs tended to grow slightly faster than wild-type NPCs (Fig. 7d). Upon differentiation of NPCs into neuronal cells, we observed that several marker genes, such as glial fibrillary acidic protein (*Gfap*) and Nestin (*Nes*), were dysregulated upon SAMD1 KO (Fig. 7e). Immunofluorescence of the differentiated cells on day 6 showed an enhanced level of Tuj1 (Tubb3)-stained neurons in the KO cells (Fig. 7f), indicating that SAMD1 KO enhances the differentiation preferentially towards neurons. This observation is consistent with the increased expression of genes related to neuronal pathways during ES cell differentiation^4^ (Supplementary Fig. S5a). Furthermore, we observed higher levels of H3K4me2 in the differentiated cells (Fig. 7f,g). This observation supports that SAMD1 deletion may impair the activity of the KDM1A histone demethylase complex during neuronal differentiation, in line with our previous observation in undifferentiated ES cells^4^. However, other indirect effects could also be the source of this observation. Combined, these data support the hypothesis that the absence of SAMD1 leads to aberrant neuronal differentiation and supports that SAMD1 is critical for proper differentiation processes.

### SAMD1 heterozygous mice fail to thrive

Finally, we assessed the consequence of a heterozygous deletion of SAMD1. H&E staining of various organs of an E14.5 heterozygous (HET) embryo was not noticeably different from that in the WT embryo (Supplementary Fig. S2), and HET mice were born alive. This less severe phenotype of the heterozygous mice, compared to the knockout mice, can likely be explained by a still relatively high expression level of SAMD1 in heterozygous cells (Fig. 1c). However, analysis of the surviving mice showed that approximately 30% of the HETs failed to survive past P21 (Supplementary Tables S2, S3), suggesting that the HET mice have distinct phenotypes.

**Figure 8:**
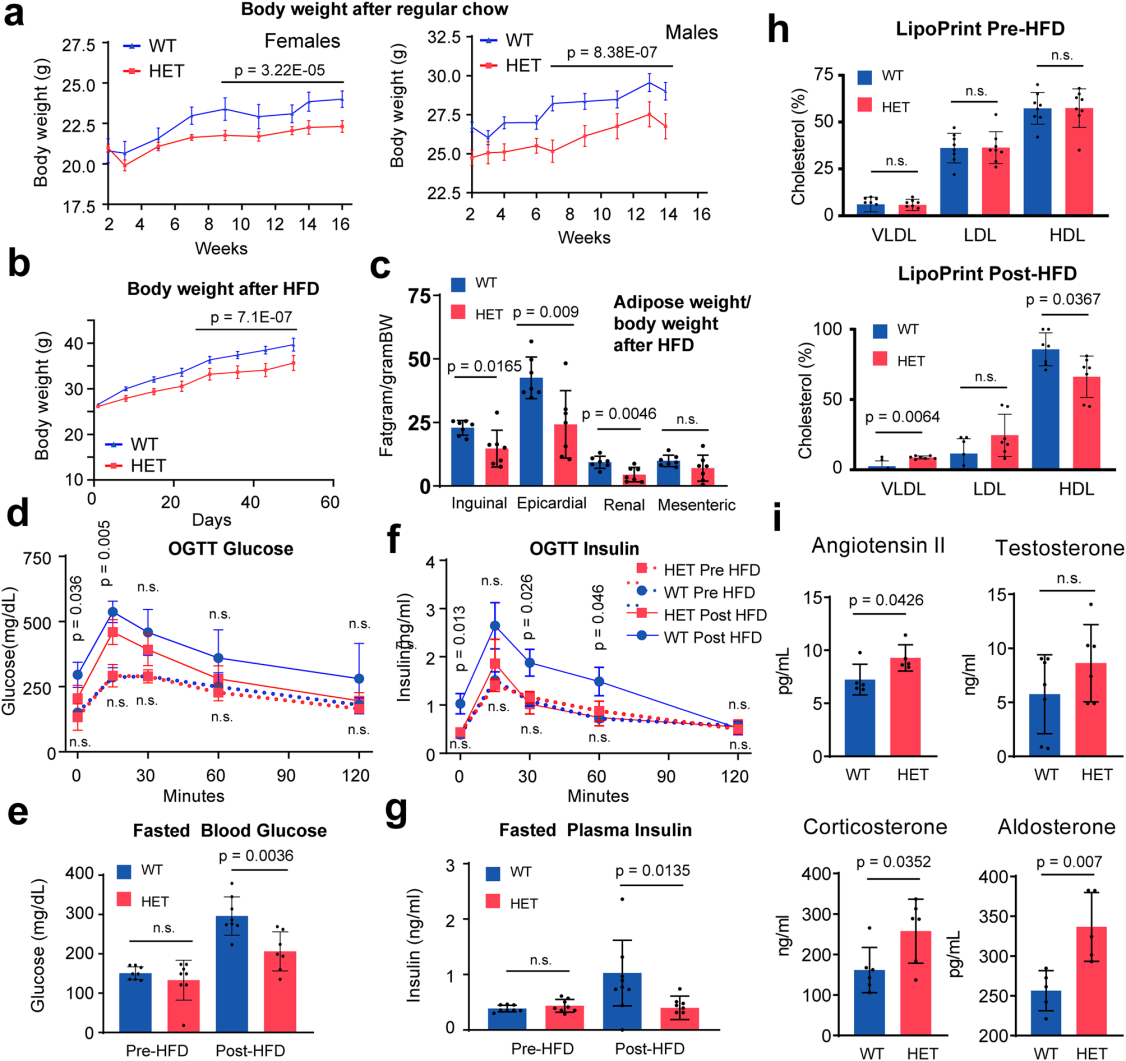
SAMD1 heterozygous (HET) mice gain less weight and have reduced adiposity, higher hormone levels, and different serum cholesterol levels. (**a**) Weight gain of WT and HET mice fed regular chow. Data are presented as the mean ± SE of at least 7 mice per condition. Significance was evaluated by a two-tailed unpaired Student’s t-test for the last 5 time points combined. (**b**) Weight gain of WT and HET mice fed regularly with high-fat diet (HFD). Data are presented as mean ± SE of at least 7 mice per condition. Significance was evaluated by a two-tailed unpaired student’s t-test. (**c**) Adipose weight upon HFD (n = 7). Data are presented as the mean ± SD of 7 mice per condition. P-value via two-tailed unpaired Student’s t-test. (**d**) Blood glucose concentration during oral glucose tolerance test, pre- or post-HFD. Data are presented as mean ± SE of at least 7 mice per condition. P-values via two-tailed unpaired student’s t-test. (**e**) Fasted Blood Glucose level pre- or post-HFD. Data are presented as the mean ± SD of at least 7 mice per condition. Significance was evaluated by a two-tailed unpaired Student’s t-test. (**f**) Serum insulin during the oral glucose tolerance test, pre- or post-HFD. Data are presented as the mean ± SE of at least 7 mice per condition. Significance was evaluated by a two-tailed unpaired Student’s t-test. (**g**) Fasted plasma insulin pre- or post-HFD. Data are presented as the mean ± SD of at least 7 mice per condition. Significance was evaluated by a two-tailed unpaired Student’s t-test. (**h**) Serum cholesterol levels before and after an HFD. Data are presented as mean ± SD of 7 mice per condition. P values via two-tailed Mann–Whitney U test. (**i**) Levels of metabolic hormones in WT and HET mice. Data are presented as mean ± SD of at least 5 mice per condition. P-values via two-tailed unpaired Student’s t-test. n.s. = not significant.

To gain a clearer picture of why some of the postnatal HET mice died, we analyzed key body measures of the surviving mice. Longitudinal body weight analysis on regular chow revealed that male and female HET mice weighed less than WT mice (Fig. 8a), suggesting a reduced ability to thrive. A high-fat diet (HFD) is commonly used to induce metabolic changes in mice. HFD (60% fat by kcal, vegetable shortening) was started at 4 weeks and continued for 15 weeks. We found that after HFD feeding, HETs weighed less than control WT mice (Fig. 8b), and fat depots from HET mice weighed approximately half as much as those from the WT mice (Fig. 8c). Consistent with what appeared to be reduced adiposity in the SAMD1 HET mice on the HFD, glucose disposal was increased in the HET mice during the oral glucose tolerance test (OGTT) (Fig. 8d) and baseline (time 0) fasted glucose levels were significantly lower in the HET mice following the HFD challenge (Fig. 8e). Insulin measurements from the blood samples collected during the OGTT found that insulin levels in the HET mice did not increase following the HFD (Fig. 8f) and were significantly lower than the corresponding levels in the WT mice (Fig. 8g).

Serum VLDL (very low-density lipoprotein) and LDL (low-density lipoprotein) were higher in the HETs, while HDL (high-density lipoprotein) was lower in the HETs (Fig. 8h). Proper steroidogenesis is essential for correct lipid metabolism^38^. Indeed, steroid hormone levels appeared to be altered in adult HETs. Corticosterone, aldosterone, and angiotensin II levels were significantly increased, and testosterone levels trended higher (Fig. 8i). Steroidogenesis and transformation of cholesterol to oxysterols occurs in many cell types^39^. It is possible that the approximately 10–15% reduction in SAMD1 expression in SAMD1 HET mice (Fig. 1c) caused or required a compensatory change in steroid expression. The lower weights and adiposity may also be related to steroidal differences. Notably, we measured large differences in the above markers between individual adult HETs. It is likely that the HETs dying prior to postnatal week 3 expressed a more severe phenotype, resulting in a failure to thrive. We did not observe any, probably because they are typically quickly eaten by the dams.

Collectively, these data suggest that although the heterozygous deletion of SAMD1 has no substantial consequences on embryogenesis, it still has an impact on the ability of the mice to develop normally after birth.

## Discussion

SAMD1 is an epigenetic regulator that plays a repressive gene regulatory role at unmethylated CpG islands^4,9^. Upon mouse ES cell differentiation, the absence of SAMD1 enhances the expression of genes involved in neuronal pathways, while factors important for metabolism and angiogenesis are downregulated (Supplementary Fig. S5)^4^, likely due to both direct and indirect effects. In HepG2 liver cancer cells, SAMD1 also strongly impacts the transcriptional network^9^. In addition to gene regulatory functions SAMD1 may also have a role in atherosclerosis^1,3^ and muscle adaptation^15^. In this study, we investigated the effects of ablation of the SAMD1 gene in mice.

Looking at the whole organism, we noted that without SAMD1 there was an absence of functional blood vessels leading to a pale appearance of the embryo by E14.5 (Fig. 1e). Similarly, RBCs, and the lack thereof, in the E14.5 KO yolk sac, placenta, and umbilical cord seem to mark failed vessels, resembling those seen in the embryo. This should not be surprising, since vessel development is similar in embryonic and extraembryonic tissue^40–42^. Given that by approximately E12.5 the definitive placenta is mature^43^ and the E12.5 embryos and extra-embryonic tissue appeared grossly normal, we conclude that embryonic lethality of the SAMD1 KO mice is unlikely to have placental defects as a primary cause. We hypothesize therefore that lack of SAMD1 causes roughly simultaneous failure of blood vessels in the embryo, the yolk sac and the placental vasculature.

Nonetheless, SAMD1 KO mice, including the extraembryonic tissues, necessarily had developed a vascular plexus, at least to the point of EC tubes. This is shown by the extent of organ development and the presence of apoptotic EC fragments in locations where vasculature would normally have been. Additionally, the observed RBCs and RBC clusters could not have arrived at the observed locations without a circulatory system to transport RBCs. The sparse mix of nucleated and enucleated RBCs in the KO demonstrated that early hematopoiesis, functional heartbeat, and vasculogenesis had occurred (Figs. 1f, 3a, 6)^44,45^. These findings suggest that in the SAMD1 KO mice at least partially functional ECs differentiated, proliferated, connected, and organized into a functional vascular plexus during the early stage of embryogenesis. However, the E14.5 KO no longer has functional vasculature. At this stage, staining for EC markers appeared to mark only necrotic cells (Figs. 3,4,5), and RBCs were seen only in small clusters and scattered broken lines suggesting that previously functional blood vessels had degraded. Failure of arteriogenesis and subsequent pruning^20^ seems a possible explanation for the termination of circulation in the SAMD1 KO mice at this time point, since that such a failure would likely also lead to collapse of angiogenesis^19,20^. Although significant research is ongoing, factors involved in arteriogenesis are still poorly understood compared to angiogenesis^36,46^, and it remains to be determined by which mechanisms absence of SAMD1 may impair vessel maturation.

The absence of functional vasculature in the entire embryo at this time point likely caused severe hypoxia and thus cessation of heartbeat. Prior to initiation of maternal-embryo circulation, nutrients and sufficient oxygen to metabolize glucose for growth is available in the uterus. Normal heartbeat starts by E8.5, providing circulating nutrients and RBCs for oxygen^47^. Cessation of circulation at any time after approximately E9 would cause hypoxia such that the heart could no longer function, and resorption would begin. The SAMD1 KO heart, liver, and lung were the correct size at about E12.5–E13.5. Obviously, organs could not have developed to this size without a functional circulatory system to provide nutrients and oxygen. At E14.5, we found only fragmented vasculogenesis-level blood vessels. RBCs were absent from most locations, and pooled in a few places near the embryo surface, meaning that circulation had ceased, causing severe hypoxia and thus, cessation of the heartbeat. The observed tissue degradation means that resorption was occurring, which is very rapid after cessation of heartbeat^48^ suggesting that the death in the E14.5 mouse occurred at approximately E13.5.

Taken together, we hypothesize that the death of the SAMD1 KO embryos is likely driven by several successive events. Possibly, arteriogenesis failed first, leading to failed vessel maturation^19^, vessel pruning^20^, and thus failing circulation. It is unlikely that this occurred simultaneously throughout the embryo, but at some point, it would prevent sufficient oxygen supply to the heart, leading to cessation of the heartbeat, an event that marks death and initiates resorption followed by the observed degradation of the embryos. Circulation failure caused insufficient oxygen and nutrient supply to internal organs, likely affecting their development. Secondary events, such as bone ossification are possibly driven by the hypoxic conditions during the failure of the circulation. Notably, the observed SAMD1 KO phenotype is markedly different from reported deletions of regulators of vasculogenesis, angiogenesis, and lymphatic development^47^. Thus, SAMD1 may modulate vessel formation in a unique fashion, which requires further clarification.

The presence of SAMD1 mRNA in the entire WT embryo and yolk sack (Fig. 1g,h, Supplementary Fig. S1) suggests that SAMD1 is relevant beyond vessel development. Thus, some SAMD1 KO phenotypes may not be related to failure of vascular development. The high expression of SAMD1 in the head, as well as the consequences of SAMD1 deletion on neuronal differentiation processes (Fig. 7), supports a role of SAMD1 during head and neuronal development (Fig. 1f). Given that undirected ES cell differentiation in the absence of SAMD1 influences multiple distinct pathways^4^, it is likely that SAMD1 is involved in additional cellular processes, which awaits further clarification. Thus, experiments that address the role of SAMD1 during other differentiation processes will be important to assess the relevance of SAMD1 for distinct lineages. Generating conditional SAMD1 knockout mice may be a suitable strategy to address the role of SAMD1 during the development of specific tissues and organs. Useful data might also be gathered from studies of SAMD1 KO mice that were embryonic lethal later in development.

Mutations that result in the absence of a gene often do not have uniform effects across a population. The mechanisms can be environmental, epigenetic, synergistic, and multigenic^49^. Variable expression and penetrance are likely explanations for the survival until as late as E18.5, of the few KOs that had heartbeats past E14.5 (Supplementary Table S1), and for the approximately 30% postnatal HET mortality by 3 weeks of age (Supplementary Tables S2 and S3). It is possible that compensating proteins, which typically have domain similarities with a knocked-out protein, allowed at least partial rescue of the developing embryo^16^. The E15.5 KO shown in Fig. 1f may be a somewhat different phenotype from the E14.5. The scarcity of blood vessels is not as extreme, but the absence of skull vault and the exencephaly in the E15.5 KO is different, and might have been caused by an “open” neural tube defect^50^.

Heterozygous SAMD1 KO mice were born alive, suggesting that here the embryogenesis was grossly normal. Likely, the relatively minor reduction of SAMD1 expression in the HET mice (Fig. 1c), possibly achieved due to compensatory effects^16^, is the underlying source for this weak phenotype. However, living HET mice had substantially increased aldosterone, corticosterone, angiotensin II, and testosterone levels (Fig. 8i), suggesting that correct SAMD1 levels are important for proper body function. Steroidogenesis and transformation of cholesterol to oxysterols occurs in many cell types^39^. Thus, it is currently unclear whether SAMD1 directly regulates hormone production pathways in cells or whether these changes are caused by compensatory effects, due to other alterations in body homeostasis. The HETs also showed reduced body weight and adiposity (Fig. 8a–c), which may be linked to steroidal changes.

This investigation aimed to present the first description of the effect of SAMD1 ablation on mouse embryonic development. Based on the E14.5 and E15.5 phenotypes, we focused on features related to vasculogenesis, arteriogenesis and neuronal differentiation. The study is therefore limited to a restricted time window of embryonic development and does not provide deeper insights into the role of SAMD1 during earlier or later time points. Given the use of a limited set of vascular markers and probes, only cells related to differentiated ECs were unambiguously identified. Therefore, it is not determined whether failure of the early vascular plexus is due to failure of migration, proliferation, differentiation, communication, or connection of necessary progenitor cells or of an unknown mechanism. Furthermore, we investigated only a restricted subset of animals and organs for the KO mice and only selected measures for the heterozygous mice. Thus, it is likely that other phenotypes or defects occur in the absence of SAMD1. Additionally, this study does not address the cellular and molecular mechanisms by which the absence of SAMD1 leads to the observed phenotypes. Specifically, it remains to be determined, which phenotypes are directly regulated by SAMD1, and which occur due to indirect effects. Given that SAMD1 is expressed in all tissues and organs (Fig. 1g,h), more research will be necessary to elucidate the role of SAMD1 in specific cellular contexts.

This is the first report on ablation of SAMD1 in mice. We observed embryonic lethality in the total knockout mice, which may be the consequence of the myriad effects stemming from the lack of appropriate vascular development. Given the wide expression of SAMD1 in most organs, numerous other processes may also be affected, potentially explaining the complex phenotype, both in the homozygous and the heterozygous mice.

## Methods

### Animal handling

Mice were bred, studied, and maintained in the Longwood Medical Research Center facility in accordance with guidelines of the Committee on Animals of the Harvard Medical School and those prepared by the Committee on Care and Use of Laboratory Animals of the Institute of Laboratory Resources, National Research Council [DHEW publication No. (NIH) FS-23]. The Pfizer Institutional Animal Care and Use Committee (IACUC) approved all of the animal procedures and protocols according to the criteria stated by the National Academy of Sciences National Research Council (NRC) publication 86-23, 1985. All methods were performed in accordance with the relevant guidelines and regulations, as well as in accordance with the ARRIVE guidelines.

### SAMD1 gene inactivation

SAMD1 knockout mice were genetically engineered by Pfizer (Groton, CT). To generate the SAMD1 targeting vector, recombineering^51^ was used to replace 2396 bp of the mouse SAMD1 (Accession ID D3YXK1) gene encompassing the 3′ 615 bp of exon 1, exons 2–4 and all of exon 5 except for the 3′ 83 bp with a neomycin phosphotransferase cassette in a 9433 bp genomic subclone obtained from a C57BL/6J BAC (RP23-128H6; Invitrogen). The vector was introduced into Bruce4 (mixed C57Bl/6N:C57BL/6J) mouse embryonic stem cells^52^ using a standard homologous recombination technique. Southern blot analysis was used to identify correctly targeted mouse embryonic stem cell clones. Chimeric mice were generated by injection of the targeted ES cells into Balb/c blastocysts. Chimeric mice were bred with C57Bl/6J mice to produce F1 heterozygotes. Germline transmission was confirmed by PCR analysis (SAMD1g1: 5′-CCAAACCCCTCTTCAGTTCA-3′; SAMD1g2: 5′-GCCGTAGCTATTCTGCCTCA-3′; dNEO2: 5′-ACATAGCGTTGGCTACCCGTGATA-3′). F1 heterozygous males and females were mated to produce F2 mice. The colony was maintained by intercrossing under specific pathogen-free conditions with unrestricted access to food and water.

### RNA and cDNA measurements for mouse experiments

RNA and cDNA measurements were performed at the Brigham & Women’s Hospital (Boston, MA). Synthesis of cDNA (ThermoScript RT-PCR System (Invitrogen Cat 11146-016)). RT-PCR was performed in a MyiQ single-color real-time PCR system (Bio-Rad Laboratories, Inc., Hercules, CA). Total ribonucleic acid (RNA) from 250,000 cells was reverse transcribed by Superscript II (Invitrogen) according to the manufacturer's instructions. Quantitative PCR was performed with SYBR green PCR mix (primers for SAMD1 from QIAGEN N.V.) and analysis performed with StepOne Software (ThermoFisher, Applied Biosystems). Levels of mRNA were normalized to glyceraldehyde 3-phosphate dehydrogenase (GAPDH) mRNA levels. cDNAs were synthesized using a High-Capacity RNA-to-cDNA™ Kit (Thermo Fisher, Catalog No. 4387406). Total 2 µg RNA/100 µl cDNA synthesis reaction and 1 µl cDNA per TaqMan reaction. GAPDH was used as an endogenous control (Applied Biosystem 4352339E. Catalogue No. 43-523-39E).

### Isolation of mouse embryonic fibroblasts

Mouse embryonic fibroblasts (MEFs) were isolated as described^53^. In short, embryos from WT, SAMD1^+/−^, and SAMD1^−/−^ mice were isolated between E12.5 and E18.5. Internal organs, heads, tails, and limbs were removed from embryos prior to treatment with trypsin to dissociate the cells. Cells were seeded into T-75 cell culture dishes in 15 ml of MEF media: KO DMEM (Life Technologies, 10829-018), 15% FBS (Life Technologies), nonessential amino acids (Life Technologies 111140-050), 2 mM l-glutamine (Life Technologies, 25030-081), 0.1 mM β-mercaptoethanol (Sigma M7522), and 0.25 mg/ml gentamicin (Life Technologies, 15710-072).

### Phenotypic studies

All behavioral, physical, hormonal, and response phenotype comparisons of SAMD1^+/+^ to SAMD1^+/−^ were performed by Caliper Life Sciences (Xenogen Biosciences, Cranbury, NJ) based on the Phenotype Pfinder platform^54^.

### Immunohistochemistry

Antibodies against CD31 and VEGFR2 were purchased from Abcam (Cambridge, UK). Immunohistochemical staining procedures were performed by Pfizer (Groton, CT) and at the Brigham & Women’s Hospital (Boston, MA). E14.5 has been established as the optimal time point for mouse developmental disorder phenotype analyses^18^. In short, tissues were collected immediately after E14.5 embryo collection, fixed in 10% buffered formalin and embedded in paraffin. Four micrometer serial sections were cut and mounted on glass slides. One section from each lesion was stained with hematoxylin and eosin (H&E). Other slices were stained for CD31 (ab28364) or VEGFR2 (ab2349) with hematoxylin counterstaining following Abcam’s protocols.

### Cell culture

E14 mESCs (E14TG2a) were provided from the lab of Jacqueline Mermoud (University of Marburg, Germany). SAMD1 KO E14 mESCs were established previously^4^. The WT and SAMD1 KO mESCs were cultured in Dulbecco’s modified Eagle’s medium (DMEM) and GlutaMAX (Gibco; 61965-026), 15% fetal calf serum (FCS) (Biochrom; S0115, Lot: 1247B), 1 × nonessential amino acids (Gibco; 11140-035), 1 × sodium pyruvate (Gibco; 11160-039), 1 × penicillin/streptomycin (Gibco; 15140-122), 10 µM β-mercaptoethanol (Gibco; 31350-010), and LIF (1000 U/ml; Millipore; ESG1107, lot: 3060038) on 0.2% gelatin-coated plates.

### Neuronal differentiation of mouse ES cells: embryoid body system

mESCs were plated at a density of 1 × 10^6^ on 0.1% gelatin-coated and dried 100 mm cell culture dishes for 3 days in mESC medium, and the medium was changed every 24 h. To start neurodifferentiation, 1 × 10^6^ dissociated ES cells were incubated on nonadhesive bacterial dishes (100 mm) for 6–8 days in mESC medium without LIF, and the medium was changed every 2 d. Embroid bodies (EBs) were collected and redistributed to 4 wells of a 6-well plate containing 2 ml differentiation medium containing DMEM (Gibco; 11960-044), 20% FCS, 2 mM l-glutamine (Gibco; 25030-081), 1 × penicillin/streptomycin, 1 × nonessential amino acids, 50 µM β-mercaptoethanol, and 0.5 µM *trans*-retinoic acid (Millipore; 554720) and incubated for 2 days. EBs from two wells each were collected and redistributed to a 100 mm cell culture dish, coated with 20 μg/ml poly-l-ornithine hydrobromide in PBS (Sigma-Aldrich; P3655; 4 h to overnight at room temperature, rinsed twice with water) and incubated for 7 days in neurobasal medium (Gibco; 21103-049) with 1 × L-glutamine, 1 × penicillin/streptomycin, and 1 × B-27 supplement (Gibco; 17504-044)^55^.

The established neural precursor cells (NPCs) were cultured in Euromed-N medium (EuroClone; ECM0883L) supplemented with 1 × N-2 (Gibco; 17502-048), 1 × l-glutamine, 1 × penicillin/streptomycin, 50 µg/ml BSA (Sigma-Aldrich; A9418), 20 µg/ml insulin (Merck; 11376497,001), 10 ng/ml EGF (Peprotech; 315–09), and 10 ng/ml FGF (Peprotech; 100-18B) on 0.1% gelatin-coated and subsequently 5 μg/ml laminin-coated (Sigma-Aldrich; L2020) six-well tissue culture plates and passaged with Accutase (Sigma-Aldrich; A6964) (adapted from^56^).

To promote the neurogenic capacity of NPCs and allow the formation of mature neurons, WT and SAMD1 KO NPCs were seeded at high density (6 × 10^5^) into gelatinized (0.1% in H2O) and poly-l-ornithine hydrobromide-coated (20 μg/ml in PBS) six-well tissue culture plates for differentiation with N2B27 medium, consisting of DMEM/F12 (Gibco; 11320-033) and neurobasal medium (1:1), with 1 × N-2, 1 × B-27, 100 µM β-mercaptoethanol and the addition of 10 ng/ml FGF (Peprotech; 100-18B) (adapted from^57^). Analysis of the cells was performed with immunofluorescence staining and RT-qPCR at chosen time points.

### RT-qPCR experiments

For RNA isolation, cells were cultivated on 6-well plates up to 80–100% confluency. RNA was prepared using the RNeasy Mini Kit (Qiagen; 74004) according to the manufacturer’s manual, including an on-column DNA digest (Qiagen; 79254). The PrimeScript RT Reagent Kit (TaKaRa; RR037A) was used to transcribe mRNA into cDNA according to the manufacturer’s manual. Samples were incubated for 30 min at 37 °C followed by 5 min at 85 °C to inactivate PrimeScript RT enzymes. Subsequently, cDNA was diluted 1:20 to be used in RT-qPCR. For analysis by real-time quantitative PCR, MyTaq Mix (Bioline; BIO-25041) was used. For gene expression analysis, values were normalized to mActb and mGapdh expression. The qPCR primers used are presented in Supplementary Table S4.

### Antibodies for neuronal studies

The following commercial antibodies were used: H3K4me2 (Diagenode; C15410035), TUJ1/TUBB3 (BioLegend; 801201), and GFAP (Dako; Z0334). The antibody for SAMD1 is custom-made. It is directed against the SAM domain of human SAMD1 (amino acids 452 to 538) and was produced as described previously^4^.

### Proliferation assay

To determine proliferation rates, cells were seeded on 6-well plates at a density of 1 × 10^5^ cells per well. Cell viability was determined at 1, 3, and 6 days after seeding using the MTT assay by adding 90 µl of 5 mg/ml thiazolyl blue ≥ 98% (Carl Roth; 4022) to each well. The medium was aspirated after 1 h, and stained cells were dissolved in 400 µl of lysis buffer (80% isopropanol, 10% 1 M HCl, 10% Triton X-100). Absorption was measured at 595 nm using a plate reader. All values were normalized to day 1 to compensate for variations in seeding density. The mean value of three biological replicates was determined.

### Immunofluorescence staining

WT or SAMD1 KO mESCs were seeded on 0.2% gelatin-coated coverslips. WT or SAMD1 KO differentiated neural precursor and neural cells were seeded on 0.1% gelatin-coated and 20 μg/ml poly-l-ornithine hydrobromide-coated coverslips Cells were fixed with 4% formaldehyde (w/v), methanol-free (Thermo Fisher Scientific; PI28906), and subsequently permeabilized with wash buffer (0.5% Triton X-100 in PBS). Blocking was performed with 10% FCS in wash buffer. Primary antibodies were diluted 1:500 in blocking solution and incubated in a wet chamber overnight at 4 °C. Three washing steps of the cells were performed before incubation with secondary antibodies, using Alexa Fluor 488 goat anti-rabbit IgG (H + L) and Alexa Fluor 546 goat anti-mouse IgG (H + L) (Thermo Fisher Scientific; A-11008 and A-11003), at a 1:2000 dilution each. Following three washing steps, the coverslips were mounted onto microscopy slides using VECTASHIELD® Antifade Mounting Medium with DAPI (Vector Laboratories; H-1200) and sealed. Microscopy was performed using a Leica DM5500 microscope, and data were analyzed using ImageJ (Fiji).

### Immunofluorescence quantification

To determine the cellular fluorescence in microscopy images using ImageJ, cells of interest were outlined and measured for their area, integrated intensity and mean gray value. Adjacent background regions were measured for the same parameters to calculate the corrected total cell fluorescence (CTCF) according to the following formula: CTCF = integrated density – (area of selected cell × mean gray value of background reading). Average CTCF values and standard deviations for 5 cells each in 3 different fluorescence microscopy images per sample are presented in a graph.

### Bioinformatics analysis

Gene expression data from mouse embryo tissues were obtained from ENCODE (https://www.encodeproject.org/, under “Mouse Development”)^24^ and from GSE45719^23^. GSEA was performed with standard settings^58^ using previously published data (GSE144396)^4^.

### Statistical analysis

Quantitative results are expressed as mean ± standard deviation of the mean (SD). Deviation from the mendelian ratio was evaluated using Chi Square tests. The significance of differences between two independently measured groups were evaluated using a two-tailed unpaired t-test. Two-way ANOVA followed by Tukey’s postdoc test was applied for multiple group comparisons. Significance of the Lipoprint analysis was evaluated using a two-tailed Mann–Whitney *U* test. The p-values were calculated using the GraphPad Prism Software. The p-values of the GSEA analysis were obtained from the GSEA software^58^. A value of p < 0.05 was considered statistically significant.

## Supplementary Information

Supplementary Information.

## Data Availability

The datasets generated during and/or analyzed during the current study are available from the corresponding authors on reasonable request. Gene expression data analyzed during the study are available from ENCODE^24^ (https://www.encodeproject.org/, under “Mouse Development”) and from the GEO repository (GSE45719^23^ and GSE144396^4^).
