# Supplementary material for: Investigation of SAMD1 ablation in mice
Source: Sci Rep. 2023 Feb 21;13:3000. doi: 10.1038/s41598-023-29779-3 (PMC9944271; doi:10.1038/s41598-023-29779-3)
Supplement: Supplementary file 1 — Supplementary Information. [file 41598_2023_29779_MOESM1_ESM.pdf]

Supplementary Information

Investigation of SAMD1 Ablation in Mice

Bruce Campbell[1,#,$,*], Lisa M, Weber[2,$], Sandra J. Engle[4,†], Terence R.S. Ozolinš[5,†], Patricia Bourassa[6,†], Robert Aiello[6,†], Robert Liefke[2,3,*]

[1] Retired
[2] Institute of Molecular Biology and Tumor Research (IMT), Philipps University of Marburg, 35043 Marburg, Germany
[3] Department of Hematology, Oncology, and Immunology, University Hospital Giessen and Marburg, 35043 Marburg, Germany
[4] Biogen, Cambridge, MA 02142, USA
[5] Queen's University, Department of Biomedical and Molecular Sciences, Kingston, Ontario K7L 3N6, Canada
[6] Cybrexa Therapeutics, Groton, CT 06340, USA

[#] Previous affiliation: Atherex Inc., Lincoln, MA 01773, USA
[†] Previous affiliation: Pfizer Inc., Groton, CT 06340, USA

[$] These authors contributed equally

* Correspondence:

Bruce Campbell
bcampbell9516@gmail.com

Robert Liefke
robert.liefke@imt.uni-marburg.de

Embryo Genotypes and Lethality Mapping						
Embryonic Day	+/+	+/−	−/−	Total N	p-value	p-value
10.5	1 (5%)	10 (56%)	7 (39%)	18	0.12	E10.5-E12.5 p=0.45
11.5	2 (13%)	7 (47%)	6 (40%)	15	0.33	
12.5	27 (23%)	61 (54%)	25 (22%)	113	0.67	
14.5	29 (30%)	53 (54%)	16 (16%)	98	0.13	E14.5-E18.5 p=0.049
16.5	2 (11%)	10 (56%)	6 (33%)	18	0.36	
18.5	16 (46%)	15 (43%)	4 (11%)	35	0.011	
Expected Mendelian ratio	1 (25%)	2 (50%)	1 (25%)			

Supplementary Table S1: Embryonic genotyping.

Embryos were genotyped between E10.5 and E18.5. The decreased total number of KO embryos from E14.5 to E18.5 ($p<0.049$) suggests embryonic lethality between E12.5 and E14.5. Chi-square calculated p-values (1:2:1 expected ratio) are shown for each embryonic day, for E10.5-E12.5 combined, and for E14.5-E18.5 combined.

Embryo Lethality Mapping when Crossing: Samd1$^{+/-}$ x Samd1$^{+/-}$						
Sex	+/+	+/−	−/−	Total N	p-value KO Mortality (1:2:1 ratio)	p-value HET Mortality (1:2 ratio)
Male (55.2%)	35 (47%)	39 (53%)	0	74	5.8×10^{-8}	0.01
Female (44.8%)	26 (43%)	34 (57%)	0	60	7.5×10^{-6}	0.01
Total M+F	61 (45.5%)	73 (54.5%)	0	134	5.1×10^{-13}	0.003
Expected Mendelian ratios	1 (25%)	2 (50%)	1 (25%)			

Supplementary Table S2: Samd1$^{+/-}$ x Samd1$^{+/-}$ crossings.

Genotyping of Samd1$^{+/-}$ x Samd1$^{+/-}$ crossings at P21. P-values for KO lethality were calculated using Chi-square and the 1:2:1 Mendelian ratio. P-values for HET lethality were calculated using Chi-square for a 1:2 Mendelian ratio.

Embryo Lethality Mapping when Crossing: Samd1$^{+/+}$ x Samd1$^{+/−}$				
Sex	+/+	+/−	Total N	p-value HET Mortality (1:1 ratio)
Male (50.9%)	69 (56%)	55 (44%)	124	0.21
Female (49.1%)	76 (63%)	44 (37%)	120	0.003
Total M+F	145 (59.5%)	99 (40.6%)	244	0.003
Expected Mendelian ratios	1(50%)	1 (50%)		

Supplementary Table S3: Samd1$^{+/−}$ x Samd1$^{+/−}$ crossings.

Genotyping of Samd1$^{+/−}$ x Samd1$^{+/−}$ crossings at P21**.** P-values for HET lethality were calculated using Chi-square for the 1:1 Mendelian ratio.

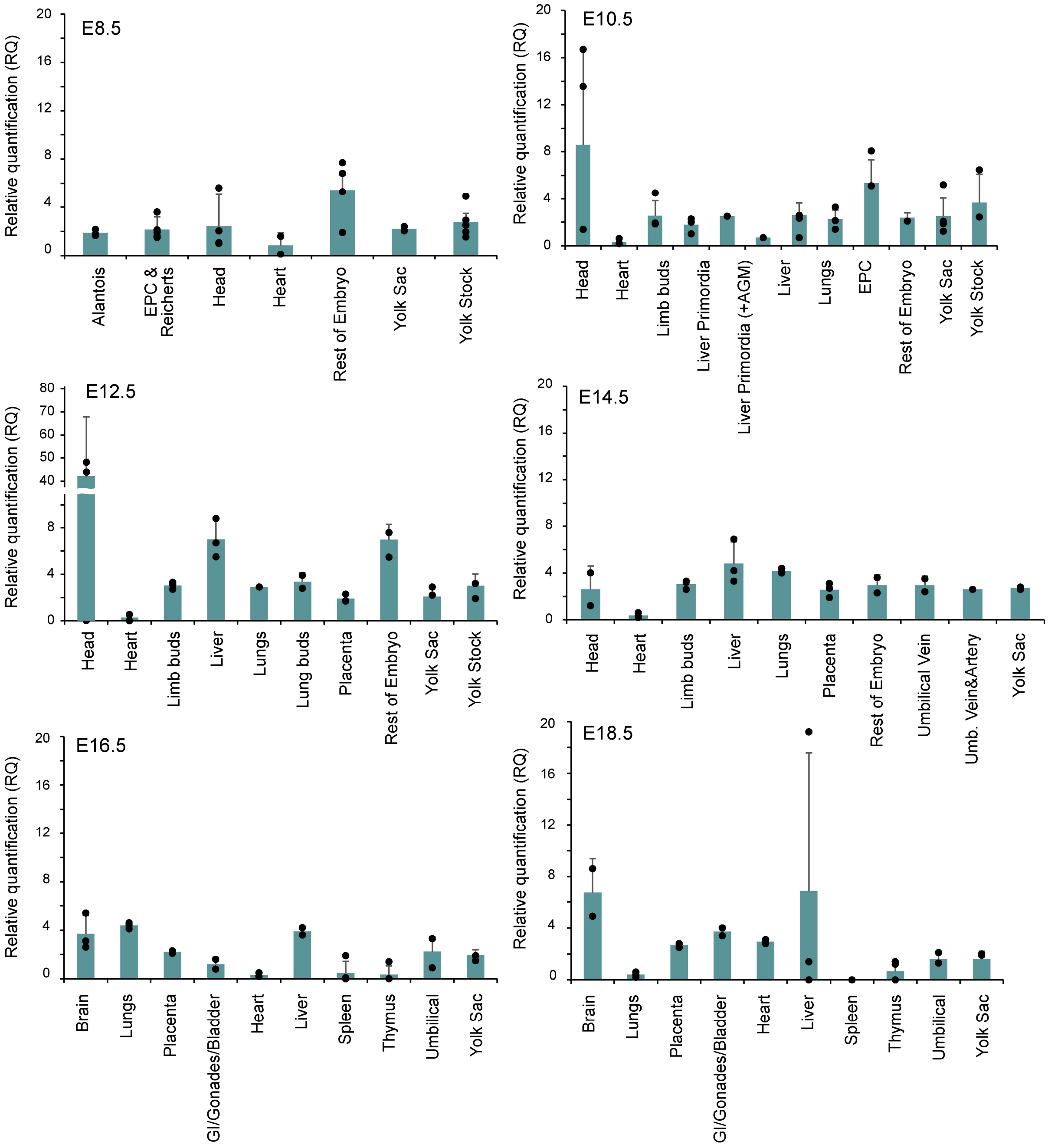

Supplementary Figure S1: Gene expression of SAMD1 during embryogenesis.

RT-qPCR analysis of mRNA levels of SAMD1 using samples from wild-type embryos at various stages. Data were normalized to *Gapdh*, and presented as relative quantification (QC) compared to the SAMD1 mRNA level from an entire E8.5 embryo. Data are presented as mean ± SD.

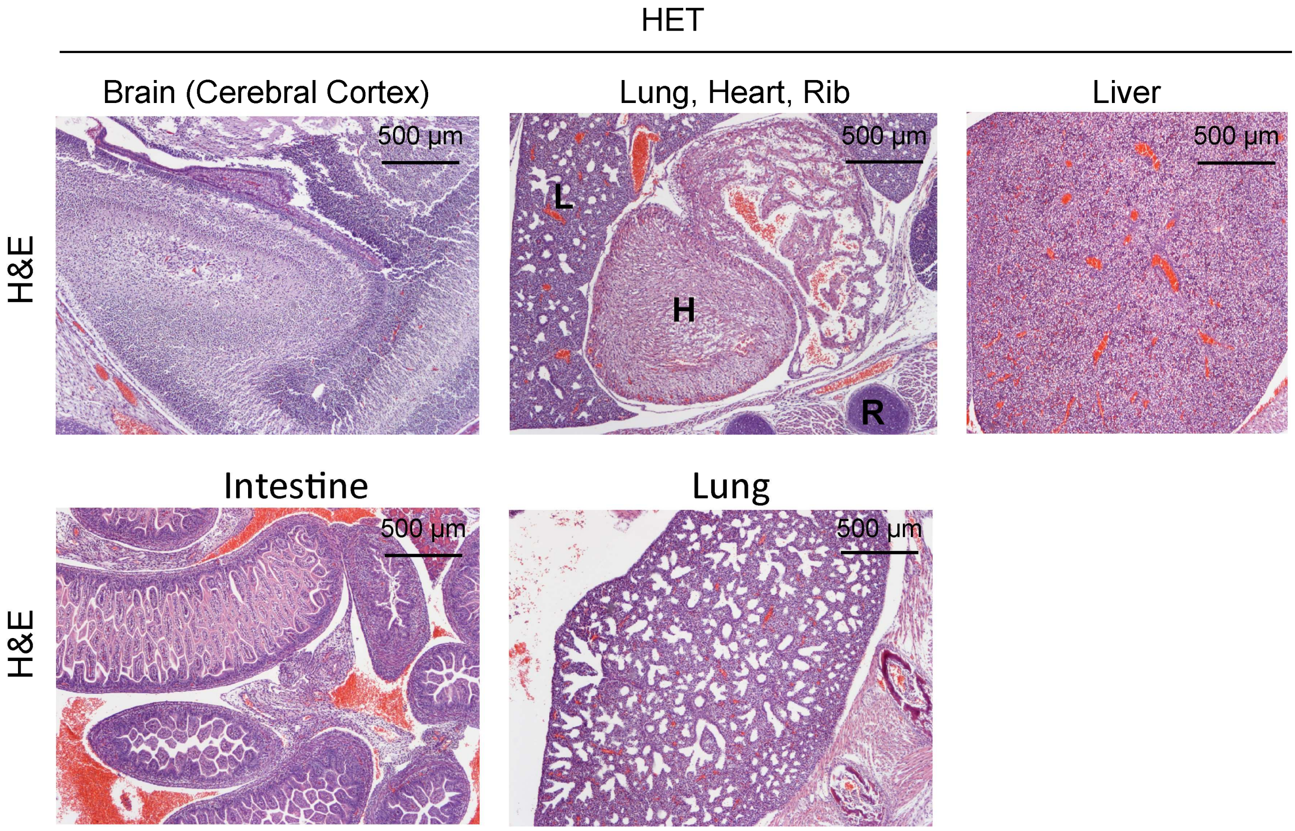

Supplementary Figure S2: Internal organs of SAMD1 HET mice are normal.

H&E staining of various organs in HET mice at day E14.5. In the upper middle panel lung (L), heart (H) and rib (R) are shown. These tissues are indistinguishable from the WT tissues shown in Figures 2 and 3.

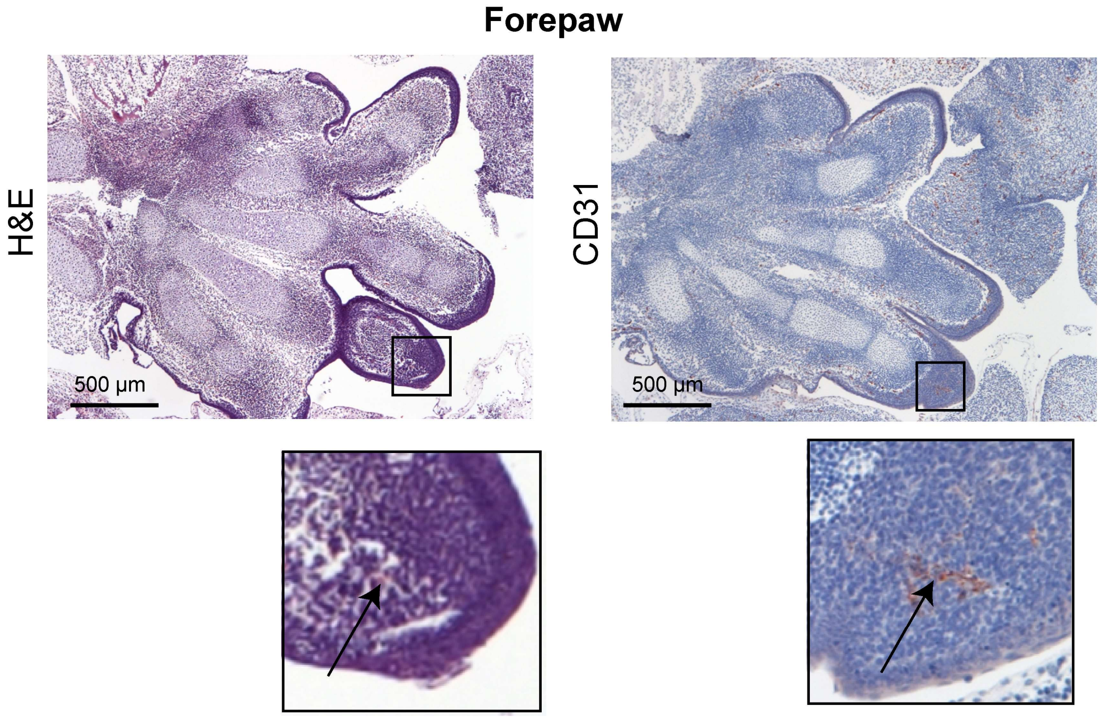

Supplementary Figure S3: H&E and CD31 staining of SAMD1 KO embryo forepaw at E14.5.

The arrows indicate a few scattered RBCs in the H&E images and misshapen ECs in a disorganized pattern in the CD31 image.

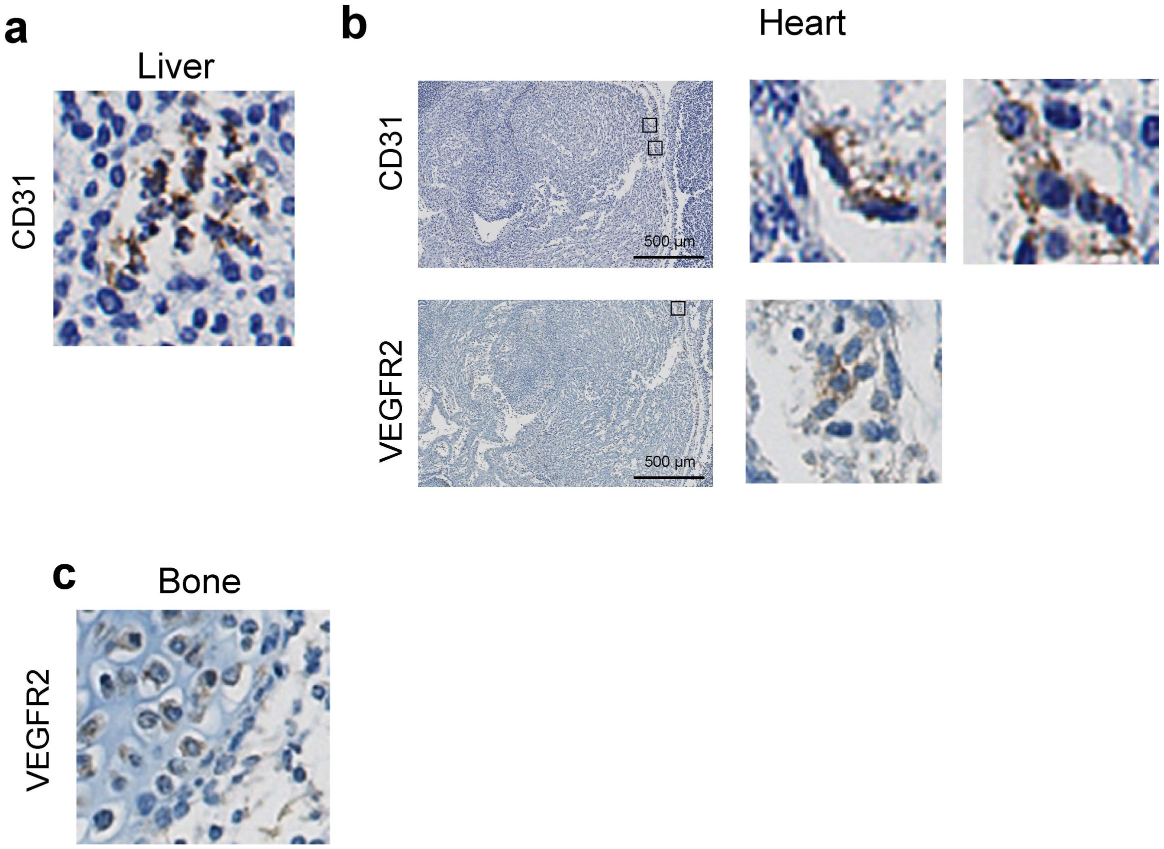

Supplementary Figure S4: Cell fragments in liver and heart of E14.5 KO embryo.

CD31 and VEGFR2 (brown) staining (counterstained with hematoxylin (blue)) in liver **(a)** and heart **(b)**, showing examples for cell fragments and phagocytosis of ECs in SAMD1 KO mice. **(c)** VEGFR2-stained chondrocytes in the bone of an E14.5 embryo.

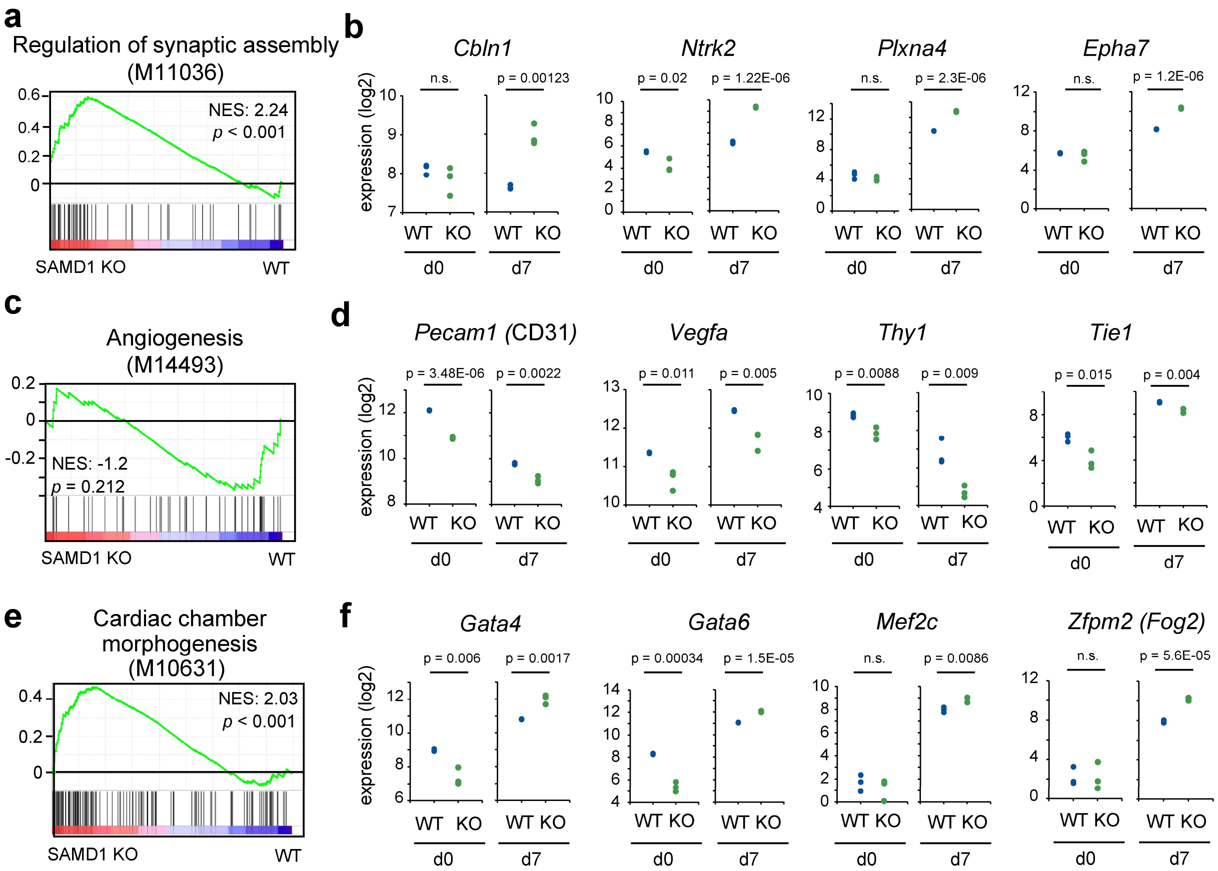

Supplementary Figure S5: SAMD1 deletion impairs multiple cellular pathways during undirected ES cell differentiation.

(a,c,e) GSEA (gene set enrichment analysis) of RNA-Seq data after 7 days of undirected ES cell differentiation[4]. **(a)** GSEA of synaptic assembly pathways. **(b)** Example dysregulated genes in differentiated SAMD1 KO mES cells related to neuronal pathways. **(c)** GSEA of angiogenesis pathways. **(d)** Examples of genes related to angiogenesis and arteriogenesis affected upon SAMD1 deletion in undifferentiated (d0) and differentiated (d7) ES cells. **(e)** GSEA of the cardiac chamber morphogenesis pathway. **(f)** Example dysregulated genes in SAMD1 KO cells related to heart development. Data from RNA-Seq. P-values two-tailed unpaired student's t-test. n.s. = not significant.

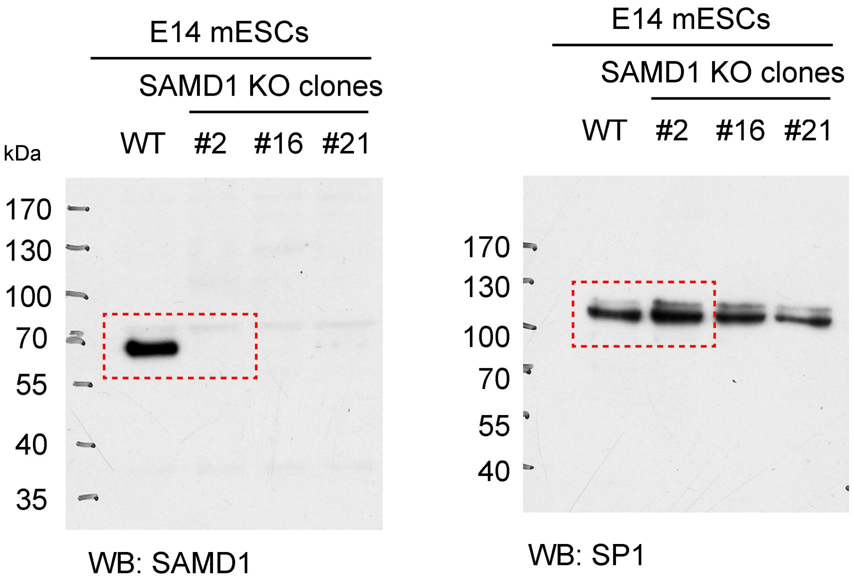

Supplementary Figure S6: Full Western blots.

Supplementary Table S4: RT-qPCR primers

Housekeeping genes	
RT_mActb_fw	GTA-CCC-AGG-CAT-TGC-TGA-CA
RT_mActb_rv	AGG-GTG-TAA-AAC-GCA-GCT-CAG
RT_mGapdh_fw	AGA-CGG-CCG-CAT-CTT-CTT-GT
RT_mGapdh_rv	GCC-TTG-ACT-GTG-CCG-TTG-AA
Target genes	
RT_mActn1_fw	GAT-ATT-GGC-AAC-GAC-CCC-CA
RT_mActn1_rv	TAA-TGA-ACC-CAG-CCA-GCC-AG
RT_mCbln1_fw	GAG-CCG-TCC-GAG-ATG-AGT-AA
RT_mCbln1_rv	CAC-CCG-TTC-AAC-ATG-AGG-CT
RT_mCdh2_fw	TGG-GAA-TCA-GAC-GGC-TAG-AC
RT_mCdh2_rv	GGT-AGT-CAT-AGT-CCT-GGT-CC
RT_mChd7_fw	CAG-ACG-TGC-TGT-TTT-CCT-CG
RT_mChd7_rv	CAG-TGA-GGA-GAC-GGT-CAA-AC
RT_mCldn11_fw	TTG-ACA-GTT-CTC-CCC-TGC-AT
RT_mCldn11_rv	GTA-GCC-AAA-GCT-CAC-GAT-GG
RT_mFoxa2_fw	GAT-GGA-AGG-GCA-CGA-GCC
RT_mFoxa2_rv	GTA-TGT-GTT-CAT-GCC-ATT-CAT-CCC
RT_mGalnt9_fw	CTC-TAC-CCC-TGT-CAT-GGC-AT
RT_mGalnt9_rv	TCT-CTA-CCT-CCA-GAC-ACC-GA
RT_mGfap_fw	AAG-GTT-GAA-TCG-CTG-GAG-GA
RT_mGfap_rv	CAC-TGC-CTC-GTA-TTG-AGT-GC
RT_mIsl1_fw	CCC-TCT-CAG-TCC-CTT-GCA-TC
RT_mIsl1_rv	GCG-TTT-CTT-GTC-CTT-GCA-CC
RT_mKlf4_fw	AAG-AGG-GGA-AGA-AGG-TCG-TG
RT_mKlf4_rv	CTG-TCA-CAC-TTC-TGG-CAC-TG
RT_mL3mbtl3_fw	CTT-GCC-TGG-GTG-TGA-AGA-AC
RT_mL3mbtl3_rv	TCC-GCA-GCT-TTG-AAC-ATC-AG
RT_mMyh6_fw	CAC-AGA-TGC-CGC-AAT-GAT-GG
RT_mMyh6_rv	GTC-GTG-CAT-CTT-CTT-GGC-AC
RT_mMyl2_fw	TTA-TTG-TTC-CAC-AGC-AGG-GGC
RT_mMyl2_rv	TGC-CCT-CGG-GAT-CAA-ACA-C
RT_mNanog_fw	CAC-AGT-TTG-CCT-AGT-TCT-GAG-G
RT_mNanog_rv	GCA-AGA-ATA-GTT-CTC-GGG-ATG-AA
RT_mNanos1_fw	GCC-TAG-TTT-AGG-TGC-GCA-AC
RT_mNanos1_rv	ACG-AGG-AAG-AAC-ACC-CTC-TC
RT_mNes_fw	GCC-TAT-AGT-TCA-ACG-CCC-CC
RT_mNes_rv	AGA-CAG-GCA-GGG-CTA-GCA-AG
RT_mNkx2-5_fw	ATT-TTA-CCC-GGG-AGC-CTA-CG
RT_mNkx2-5_rv	GCT-GTC-GCT-TGC-ACT-TGT-AG
RT_mNppa_fw	GCT-TCG-GGG-GTA-GGA-TTG-AC
RT_mNppa_rv	GAG-GCA-AGA-CCC-CAC-TAG-AC
RT_mNrp1_fw	AGC-TTC-GGA-CGT-TTT-CAC-CT
RT_mNrp1_rv	GGA-AGT-CAT-CAC-CTG-TGC-CA
RT_mNrp2_fw	TGC-AGG-TGA-GGA-TTT-TAA-AGA-TGA
RT_mNrp2_rv	CAG-GTG-CAG-TAA-AGG-AGG-AGG
RT_mOct4_fw	TGG-AAA-GGT-GTT-CAG-CCA-GA

RT_mOct4_rv	CCT-CAC-ACG-GTT-CTC-AAT-GC
RT_mOlig1_fw	TGA-ATC-CCA-CCT-GTT-TAG-AGC-C
RT_mOlig1_rv	CGA-TGC-TCA-CGG-ATA-CGA-GAA-TAG
RT_mPax3_fw	TCC-CAT-GGT-TGC-GTC-TCT-AAG
RT_mPax3_rv	CTC-CAC-GTC-AGG-CGT-TGT-C
RT_mPax7_fw	TGG-GGT-CTT-CAT-CAA-CGG-TC
RT_mPax7_rv	ATC-GGC-ACA-GAA-TCT-TGG-AGA
RT_mPth2_fw	GAG-ACC-TGC-CAG-ATG-TCC-AG
RT_mPth2_rv	CTG-CAT-GTA-AGA-GTC-CAG-CC
RT_mSamd1_fw	CTG-ATG-GGA-CAC-CTT-TTG-GC
RT_mSamd1_rv	GTG-AGC-ACA-TCT-GTC-CGT-TG
RT_mSfmbt1_fw	CCA-ACA-AGA-GAG-ATG-CCC-AG
RT_mSfmbt1_rv	GTG-GGA-AGG-GTA-AGG-AGC-AA
RT_mSox2_fw	AGG-AGA-GAA-GTT-TGG-AGC-CC
RT_mSox2_rv	TCT-GGC-GGA-GAA-TAG-TTG-GG
RT_mTbx5_fw_2	CCC-CCT-GTA-CAG-AGC-GAG-AAT-A
RT_mTbx5_rv_2	GGT-CGT-CTG-CGG-GAA-CAA-TA
RT_mTnnt2_fw	GTG-TGC-AGT-CCC-TGT-TCA-GA
RT_mTnnt2_rv	GCA-CCA-AGT-TGG-GCA-TGA-AG
RT_mTtn_fw	ACC-CTT-TTT-GGG-CAC-TCC-TG
RT_mTtn_rv	CTG-TTG-GCT-TTA-GTC-ACG-GC
RT_mTubb3_fw	TAG-ACC-CCA-GCG-GCA-ACT-AT
RT_mTubb3_rv	GTT-CCA-GGT-TCC-AAG-TCC-ACC
